# Conserved molecular properties of dissolved organic matter during Weddell Sea Deep Water formation

**DOI:** 10.1038/s41467-026-79040-4

**Published:** 2026-11-10

**Authors:** Gonzalo V. Gomez-Saez, Sarah K. Bercovici, Marit Renken, Sahithya P. B. Vemulapalli, Andreas Rogge, Hendrik Grotheer, F. Alexander Haumann, Moritz Holtappels, Thorsten Dittmar

**Affiliations:** 1 Alfred Wegener Institute Helmholtz Centre for Polar and Marine Sciences (AWI), Bremerhaven, Germany; 2 MARUM Center for Marine Environmental Sciences, University of Bremen, Bremen, Germany; 3 Ludwig-Maximilians-Universität München, Munich, Germany; 4 GeoBio-Center, Ludwig-Maximilians-Universität München, Munich, Germany; 5 Institute for Chemistry and Biology of the Marine Environment (ICBM), School of Mathematics and Science, Carl-von-Ossietzky Universität Oldenburg, Oldenburg, Germany; 6 Marine Biogeochemistry, National Oceanography Centre, Southampton, UK; 7 Institute of Environmental Physics, University of Bremen, Bremen, Germany; 8 Helmholtz Institute for Functional Marine Biodiversity (HIFMB), University of Oldenburg, Oldenburg, Germany; 9 Max Planck Institute for Marine Microbiology (MPI), Bremen, Germany

**Keywords:** Carbon cycle, Marine chemistry, Marine chemistry, Carbon cycle

## Abstract

Antarctic Bottom Water (AABW) is decreasing in volume due to climate change with potential consequences for abyssal carbon storage, where carbon is locked away from the atmosphere for centuries. Understanding the formation and export of dissolved organic matter (DOM) on the Antarctic shelf is therefore essential to determine how organic carbon enters this deep-sea reservoir. Here, we investigate solid-phase extractable DOM (SPE-DOM) transformations in the Weddell Sea, where half of AABW forms. Molecular and structural properties of SPE-DOM remain unchanged during dense-water formation. Shelf processes minimally affect exported organics into the deep-sea, likely due to remineralization to inorganic carbon enhanced by long residence times on the shelf. Consequently, the dominant SPE-DOM signature in Weddell Sea Deep Water (WSDW) is established before dense shelf waters mix with warmer off-shelf waters. We estimate that 0.155 ± 0.03 PgC are transferred annually from the Weddell Sea to AABW, and that its molecular composition is expected to remain conserved despite ongoing climate-driven weakening of AABW.

## Abstract (web summary)

The study reveals that molecular properties of dissolved organic matter are conserved during dense-water formation in the Weddell Sea, showing that Antarctic shelf processes minimally affect the dissolved organics exported into the deep ocean.

## Introduction

Antarctic Bottom Water (AABW) is the coldest, densest, and most voluminous water mass in the global ocean^1^. It is derived from dense shelf waters formed along the Antarctic continental shelf, caused by heat loss and brine rejection during sea ice formation and by cooling during the circulation under large ice shelves^2,3^. Once formed, dense shelf water overflows from its formation sites on the shelf into the deep ocean, and mixes with the relatively warm and saline deep waters to form AABW. AABW accounts for 30–40% of the total ocean volume ^4^ and likely plays a vital role in Earth´s climate system on long time-scales by sequestering carbon and nutrients into the abyssal ocean, where they remain out of contact with the atmosphere for centuries^5^. However, in recent decades, AABW has warmed, freshened, and decreased in volume^6,7^, suggesting a large-scale shift in Southern Ocean abyssal ocean ventilation^1^. This warming alone contributes about 10 to 14% to the total increase in global ocean heat content^1,8^. Formation of the precursors of AABW takes place on the Antarctic shelf, a region known for its high biological productivity^9,10^. Despite its key role in deep ocean circulation and long-term climate regulation, the carbon pools in AABW and its precursors remain undersampled in both time and space compared to other major ocean water masses^1,3^.

At dense-water formation sites, carbon cycling processes, such as the production, distribution, and turnover of marine dissolved organic matter (DOM), are essential to understand how organic carbon enters the deep-sea reservoir^11^. Dissolved organic carbon (DOC) represents the largest pool of marine organic carbon and rivals the CO_2_ standing stock in the atmosphere^12^. In terms of carbon inventory, 628 to 642 PgC^12,13^ is considered recalcitrant DOC, persisting in the deep ocean for millennia^14^. DOC can only be quantitatively relevant as an export mechanism if it accumulates^15^. However, marine DOC, as part of the DOM pool, is among the most complex organic mixtures known^16^, and the response of the DOM pool to surface ocean processes is not only expressed by the amount of DOC but also by its molecular composition^17,18^. Ultrahigh-resolution Fourier transform ion cyclotron resonance mass spectrometry (FT-ICR-MS) has become widely used for fast, untargeted and comprehensive molecular characterization of solid-phase extractable DOM (SPE-DOM)^18^. Although solid-phase extraction DOC (SPE-DOC) recovers on average 43% of total DOC in marine samples^19^, FT-ICR-MS identifies tens of thousands of molecular formulas per DOM sample, representing hundreds of thousands of compounds^16^. Despite this molecular-level resolution, it remains unclear whether dense-water formation, particularly in the Antarctic region, preserves the molecular composition of SPE-DOM through physical mixing, or whether shelf biogeochemical processes leave a detectable molecular imprint on the DOM exported to the abyss.

The Weddell Sea is the largest of four known key sites responsible for the export of dense shelf waters^20^, accounting for up to 50% of AABW production^21,22^. It is clearly affected by climate change, with a 30% decline in Weddell Sea Bottom Water (WSBW) volume since 1992^22^. A major fraction of the dense-water masses forms on the southern Weddell Sea shelf^23^. These water masses have an average residence time of 6 to 12 years on the continental shelf^24^. This is remarkably longer than on the Ross Sea shelf (<3.6 years^25^), which is the second largest shelf of Antarctica, or across Arctic Ocean continental shelves (3.5 ± 2 years^26^). Prolonged residence time of dense shelf waters, together with intense seasonal primary production and biogeochemical processing on the Weddell Sea shelf^27,28^, suggests that newly produced organic matter might transform before it is exported to greater depths.

**Fig. 1:**
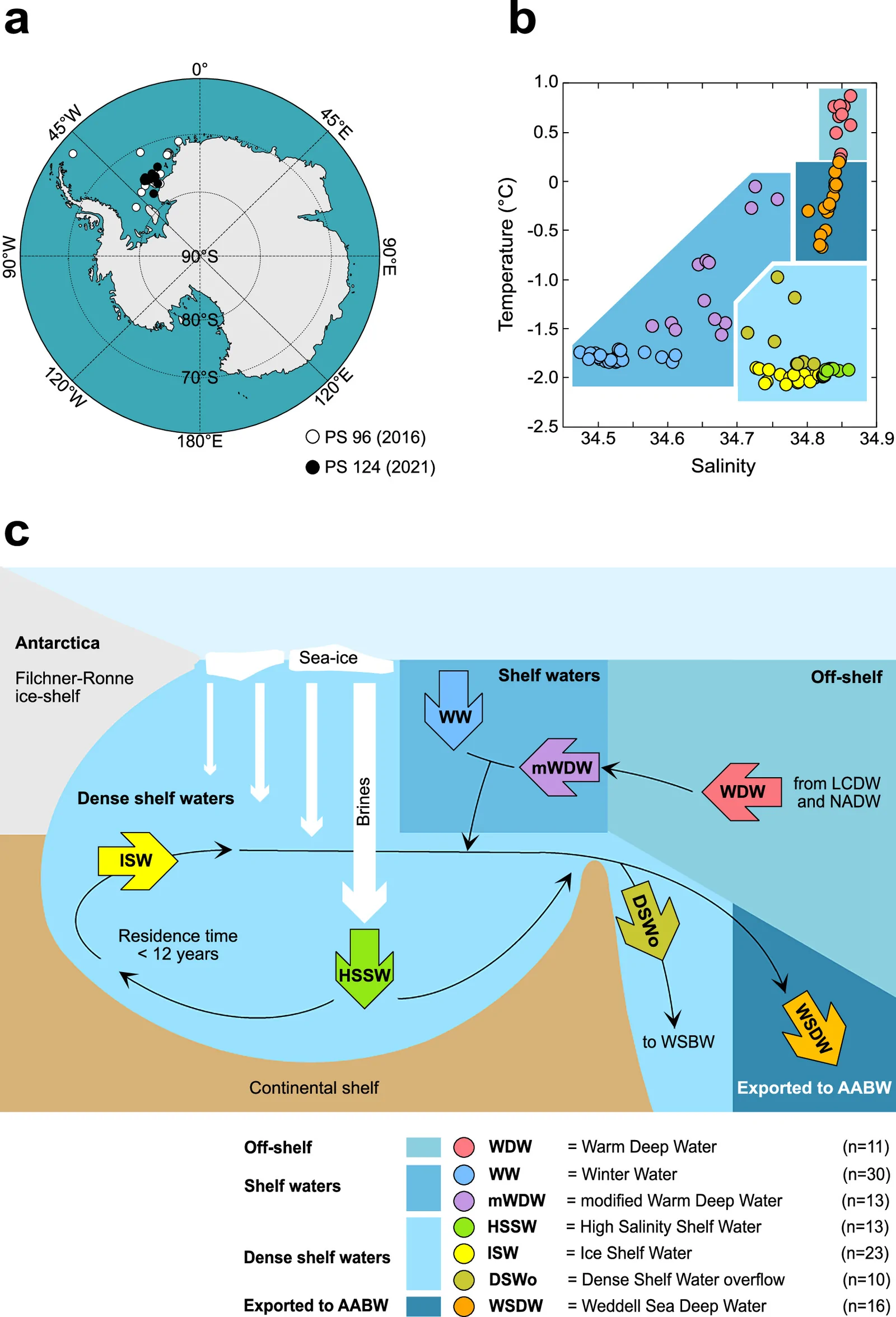
Study site and water masses involved in Weddell Sea Deep Water (WSDW) formation. **a** Water samples for this study were obtained from two different cruises to the Weddell Sea onboard the icebreaker Polarstern in 2016 (PS96, white, 19 stations) and 2021 (PS124, black, 18 stations). **b** 116 water samples from seven water masses involved in WSDW formation were defined based on water mass properties following^57–60^ (Table S1). **c** Diagram of the seven water masses involved in WSDW formation: (a) Off-shelf, warm waters: Warm Deep Water (WDW, red, *n* = 11); (b) Shelf waters: Winter Water (WW, blue, *n* = 30), modified Warm Deep Water (mWDW, purple, *n* = 13); (c) Dense shelf waters: High Salinity Shelf Water (HSSW, green, *n* = 13), Ice Shelf Water (ISW, yellow, *n* = 23), Dense Shelf Water overflow (DSWo, brown, *n* = 10); and (d) exported to AABW: Weddell Sea Deep Water (WSDW, orange, *n* = 16). Other abbreviations in the figure are: WSBW: Weddell Sea Bottom Water, LCDW: Lower Circumpolar Deep Water, and NADW: North Atlantic Deep Water. Detailed water mass classification is explained in Table S1.

**Fig. 2:**
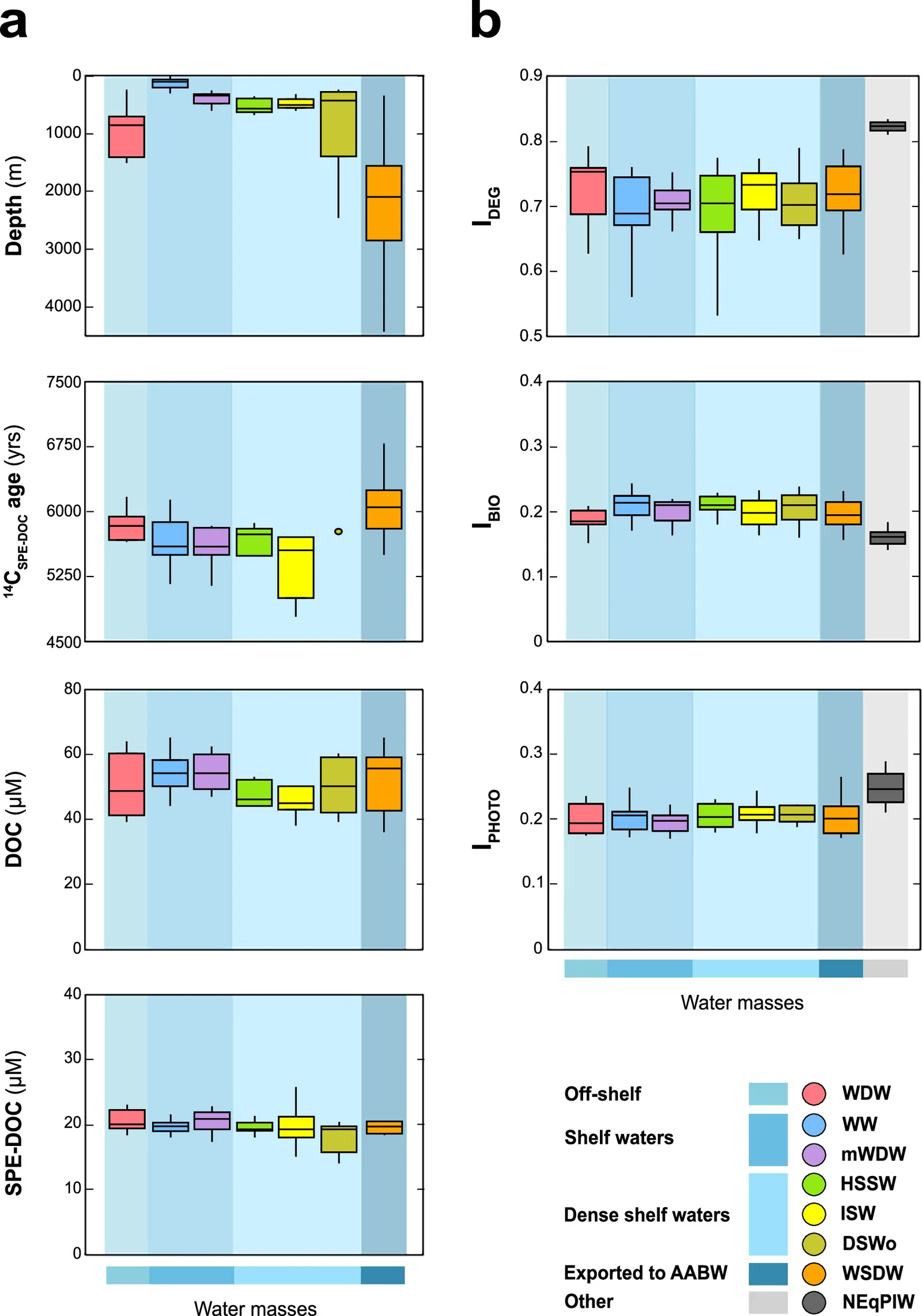
Mean values of several DOM properties across water masses indicate minimal impact of shelf processes on organic carbon export to the deep ocean. Boxplots differentiating per water mass: **a** Water depth, apparent ^14^C_SPE-DOC_ age of the solid-phase extractable fraction, dissolved organic carbon (DOC) and solid-phase extractable dissolved organic carbon (SPE-DOC); **b** Molecular proxies based on FT-ICR-MS data to assess the molecular degradation (I_DEG_^36^), microbial (I_BIO_^11^) and photochemical alterations (I_PHOTO_^11^) of DOM in FT-ICR-MS studies. **b** also includes the reference standard sample (North Equatorial Pacific Intermediate Water, NEqPIW; dark gray). More information on all other measured parameters can be found in Table S2, S4, and Data S2. Color codes are the same as in Fig. 1c.

In this study, we investigate DOM directly at the remote dense-water formation sites on the southern shelf of the Weddell Sea, tracking the organic carbon composition along successive water mass transformation pathways. These transformation processes involve a series of distinguishable water masses, which are briefly outlined in Fig. 1 and explained in more detail in “Methods”. During two expeditions with the research icebreaker Polarstern in 2016 (PS96^29^) and 2021 (PS124^30^), we collected a total of 178 water samples from 38 stations at the Weddell Sea Deep Water (WSDW) formation sites. Based on water mass properties, we differentiate 116 water samples from the surface to up to 4433 m depth, belonging to seven water masses involved in WSDW formation, the precursor of AABW (Figs. 1, 2a, S1; Table S1). Organic carbon transformations during WSDW formation are investigated by combining physicochemical and quantitative DOC analyses with molecular SPE-DOM characterization via ultrahigh-resolution mass spectrometry (FT-ICR-MS), nuclear magnetic resonance spectroscopy (^1^H-NMR), and radiocarbon (^14^C_SPE-DOC_) dating.

## Results and Discussion

### Aged, recalcitrant SPE-DOM predominates everywhere in the Weddell Sea

**Fig. 3:**
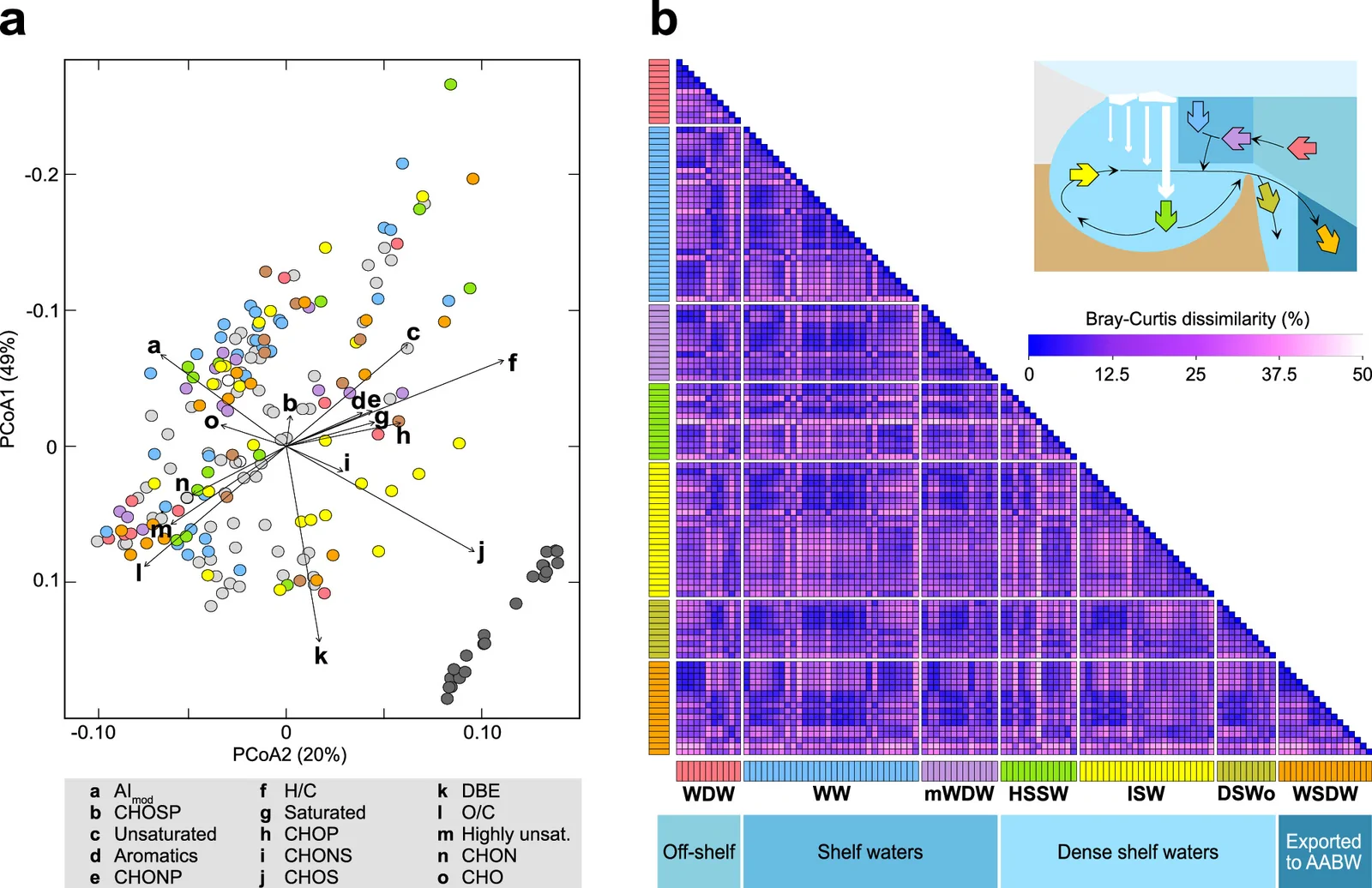
Multivariate statistics showing that molecular DOM properties detected by FT-ICR-MS were conserved during WSDW formation. **a** Principal Coordinate Analysis (PCoA) of DOM molecular formulas identified by FT-ICR-MS, including NEqPIW (dark gray, *n* = 22). The PCoA is based on the Bray-Curtis dissimilarities. Arrows include the significantly correlated molecular parameters from all water samples (*n* = 178). The samples not included in the seven water masses involved in WSDW formation are colored in light gray. **b** Bray-Curtis dissimilarity analysis of FT-ICR-MS samples from the seven water masses involved in deep-water formation (*n* = 116) is displayed as a heatmap. This dissimilarity analysis considers the relative signal intensities of molecular formulas detected by FT-ICR-MS, with white representing 50% dissimilar and blue representing 0% dissimilar (the same). Bray-Curtis matrix including all water samples (*n* = 178) is presented in Fig. S2 and all sample names and extra information are presented in Data S1.

**Fig. 4:**
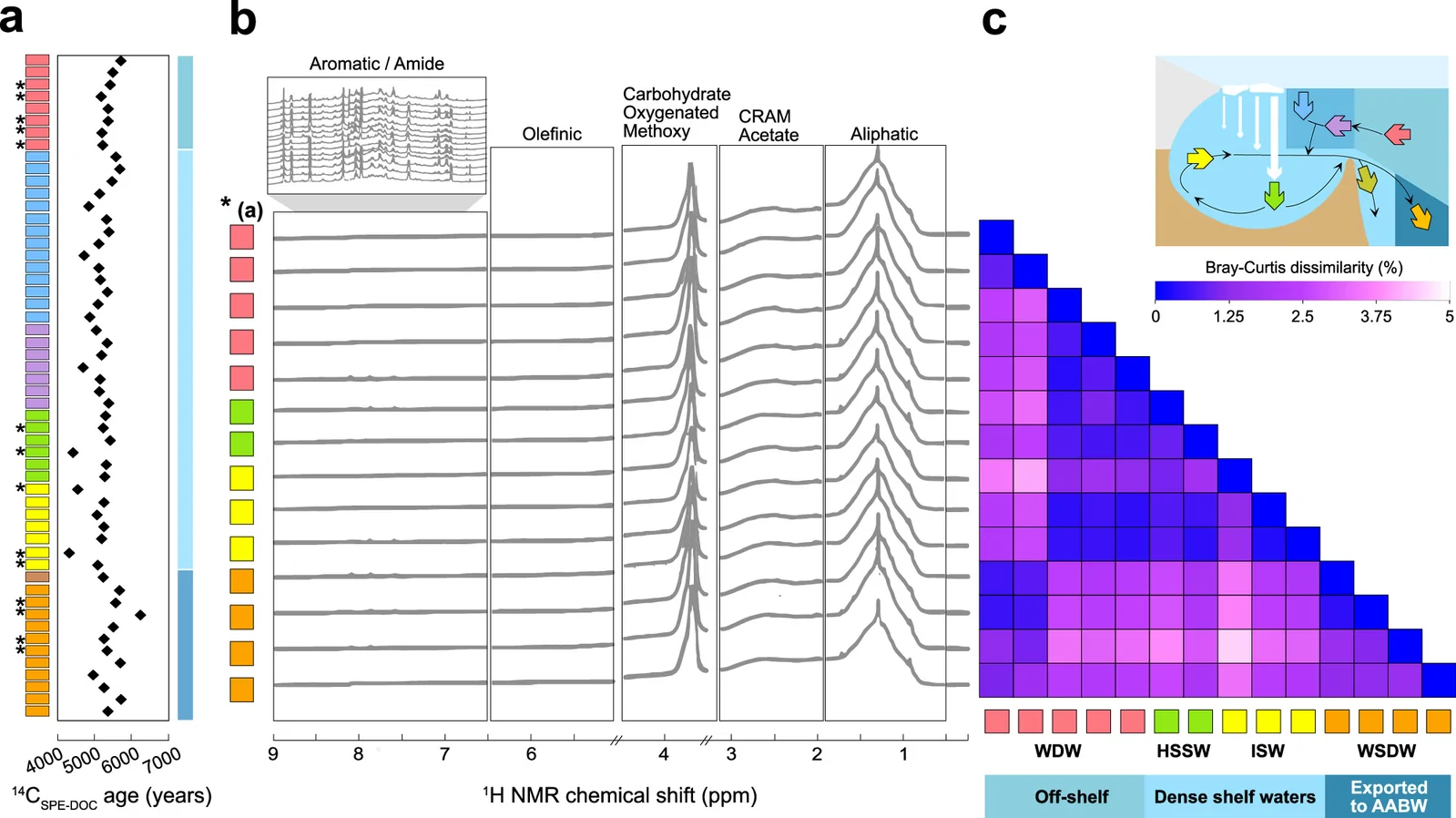
Highly similar radiocarbon ^14^C_SPE-DOC_ age and structural ^1^H-NMR properties across Weddell Sea water masses involved in WSDW formation. **a** Apparent radiocarbon ^14^C_SPE-DOC_ age of analyzed solid-phase DOM extracts carried out with the EA-GIS AMS setup. Selected samples for ^1^H-NMR are marked with asterisks. The color code relates to the water masses (see legend and Fig. 1c). **b** One-dimensional (1D) ^1^H NMR spectra of 14x samples from four different water masses (WDW, HSSW, ISW and WSDW). Each spectrum is color-coded according to its corresponding water mass. Structural group assignments are indicated by boxes as follows: Aromatic and Amide (9.0–6.5 ppm), Olefinic (6.5–5.3 ppm), Carbohydrate, Oxygenated and Methoxy (4.5–3.2 ppm), CRAM and Acetate (3.2–1.9 ppm), and Aliphatic (1.9-0.5 ppm). The inset shows a zoomed-in comparison of the Aromatic and amide regions. **c** Bray-Curtis dissimilarity analysis of ^1^H-NMR spectra. Note that the color scale differs from the other Bray-Curtis matrixes in the manuscript (Fig. 3b, S2). Because all dissimilarities were below 5%, the color scale has been adjusted to range from 0% dissimilarity (blue) to 5% dissimilarity (white).

Our findings consistently showed that SPE-DOM composition across Weddell Sea water masses involved in deep-water formation was highly similar, both at the molecular formula level (FT-ICR-MS; Figs. 2, 3) and in structural characteristics (^1^H-NMR; Fig. 4). For FT-ICR-MS and ¹H-NMR, samples had to be concentrated and desalted. Solid-phase extraction (SPE) with modified styrene divinylbenzene polymer sorbents (PPL) is the most efficient extraction method to date^19,31^. Our SPE-DOC recovery (34%; Fig. 2a; Table S2) was within the range of previous SPE extraction efficiencies for Antarctic seawater DOC (14 – 42%^32,33^). Compared to total DOM, the SPE-DOM analytical window selectively captures low molecular weight DOM^34^ likely enriched in semi-labile and recalcitrant compounds^34,35^, high degradation indices^33,35,36^ and low organic nitrogen content^34^. In our study, we focused on this fraction of DOM for molecular, isotopic, and radiocarbon characterization and combined the SPE-DOM with physicochemical and DOC measurements obtained prior to the extraction.

Molecular properties of SPE-DOM were conserved along the water mass transformation pathway: (a) from the flow of Warm Deep Water (WDW) onto the shelf as modified WDW (mWDW); (b) the transformation into dense shelf waters (HSSW and ISW) while circulating on the continental shelf; and (c) to the export from the shelf feeding into the WSDW, the precursor of AABW (Figs. 1−3). SPE-DOM from the seven investigated water masses (Fig. 1c) was highly similar at the molecular level (Fig. 3). We used FT-ICR-MS as a highly sensitive tool to detect differences between water masses^37,38^. We identified a total of 9107 molecular formulas, with an average of 3462 ± 865 formulas per sample (Fig. 3, S2; Table S2; Data S1, S2). Furthermore, we statistically assessed molecular similarity between samples using the Bray-Curtis approach (e.g., refs. ^39,40^). Molecular Bray-Curtis dissimilarity between samples was 15 ± 6% (Fig. 3b) with slight differences in molecular composition related primarily to saturation, oxygenation, and sulfur content (Fig. 3a, S3). However, these and other molecular properties did not differ systematically among Weddell Sea water masses (Welch-ANOVA; Fig. 3a; Table S3). In contrast, the deep-sea DOM reference sample from North Equatorial Pacific Intermediate Water (NEqPIW; *n* = 22) was molecularly different from all Weddell Sea samples (Fig. 3a). Further structural quantitative analysis using ¹H-NMR spectroscopy provided an orthogonal view on molecular structures compared to FT-ICR-MS^35,41^ (Fig. 4b, c). Such holistic quantitative structural molecular information is not available with any other analytical technique, but is crucial to address our research objective of tracking the organic carbon composition along successive water mass transformation pathways. Overall, the ^1^H-NMR analysis in selected samples from different water masses revealed even lower dissimilarities (Bray-Curtis: <5%; *n* = 14; Fig. 4c) and showed no consistent molecular differences in DOM across water masses.

Radiocarbon dating of SPE-DOM samples revealed the predominance of aged, presumably recalcitrant DOM in all water masses of the Weddell Sea (Figs. 2a, 4a). Our results showed that the mean Δ^14^C_SPE-DOC_ value of all samples was −510 ± 22 ‰ (F14C = 0.4907 ± 0.0213; *n* = 54; Table S2), resulting in apparent ^14^C_SPE-DOC_ ages of 5726 ± 346 years (Figs. 2a, 4b; Data S1). This age is considered ‘apparent’ because DOM is a mixture of fractions of various ages and turnover times^42^. In our samples, the WSDW exported from the shelf was slightly older than the average (6050 ± 336 years; Figs. 2a, 4a; Data S1) and reached a maximum of 6792 years in the basin (Fig. S1, S3; Data S1). On the contrary, the youngest Δ^14^C_SPE-DOC_ values were detected in dense shelf waters (Δ^14^C_SPE-DOC_ ISW = −495 ± 24‰), accounting on average to 5420 ± 372 years of apparent age (Figs. 2a, 4a; Table S2). The older ^14^C_SPE-DOC_ ages of WSDW may reflect variability in deep-water mass sources within the circumpolar current, long-time recirculation within the Weddell Gyre, or the contribution of pre-aged DOM from sedimentary sources. The sedimentary input should, however, be accompanied by an increase in DOC concentration in deeper samples close to the benthos^43^, but this was not supported by our findings, where depth-distribution of DOC and SPE-DOC showed no trends (Fig. 2a; Table S2).

Overall, the ^14^C age range of SPE-DOC in the Weddell Sea was comparable to that of bulk DOM in the deep waters of the Southern Atlantic and Pacific oceans. The apparent ^14^C-age of DOM in the deep South Atlantic Ocean is within the range of 5000 years, while in the deep South Pacific it reaches 6000 years^44^. The Southern Ocean DOC exhibits relatively small vertical radiocarbon gradients compared to lower-latitude oceans. Surface waters south of the Polar Front contain DOC with Δ^14^C values of approximately −400 to −430‰ (corresponding to radiocarbon ages of ~4000–4800 years), while deep waters there typically range from −490 to −520‰ (~5400 – 6000 years old)^45–47^. Thus, despite the deep DOC pool being among the oldest in the ocean, the difference between surface and deep DOC is only ~60–120‰, or approximately 1000–1500 radiocarbon years. This relatively small gradient reflects the rapid vertical mixing of surface and deep waters compared to lower-latitude regions. In the upper mixed layer of the ocean at lower latitudes, DOM is typically much younger due to the addition of modern radiocarbon into labile DOM^44,48^. In the Weddell Sea, ^14^C-age of SPE-DOM showed no decrease toward surface waters, and SPE-DOM molecular composition remained unchanged with depth (Figs. 2a, 4a). Taken together, these observations suggest little to no net input of labile carbon to SPE-DOM, despite the productive surface waters at the study site^9,10^. This might be related to a fast and efficient remineralization of freshly produced, younger DOC into the dissolved inorganic carbon (DIC) pool. Even further away from the actual deep-water formation sites, there is no evidence for the addition of labile carbon to DOM in deep waters of the Weddell Sea (Figs. 2a, 4a), as shown previously^33^. This observation is consistent with the observed high degree of degradation throughout the water column.

### Shelf processes minimally affect DOM export to the abyssal ocean

There was no significant difference in DOC and SPE-DOC concentration across any of the seven water masses involved in WSDW formation (Welch-ANOVA; Fig. 2a; Table S2–S4). Consistently, molecular methods demonstrated that shelf surface processes in the southern Weddell Sea did not significantly influence the DOM exported with WSDW, which ultimately incorporates into AABW. Molecular proxies have been developed for FT-ICR-MS data to assess the degradation state of marine DOM (I_DEG_)^36^, as well as microbial (I_BIO_) and photochemical (I_PHOTO_) alterations of DOM, typical for processes occurring in the sea surface mixed layer^11^. In our study, these indices showed very little variability across the samples (I_DEG_ = 0,71 ± 0.05; I_BIO_ = 0.20 ± 0.03; I_PHOTO_ = 0.20 ± 0.03; Fig. 2b) and no significant and consistent variance among the seven water masses (Welch-ANOVA; Fig. 2b; Table S3). I_DEG_ was within the same range as previously reported for surface waters south of the Polar Front^33^, even far away from areas of deep-water formation. This high degree of degradation is exceptional for DOM in surface waters and is likely associated with the rapid vertical mixing of surface and deep waters south of the Polar Front^49^. While I_DEG_ should not be interpreted as a direct measure of process-specific DOM degradation, but rather as a molecular proxy that has been shown to correlate with DOM radiocarbon age, its invariance across the studied water masses is consistent with the broader conclusion from the FT-ICR-MS, ^1^H-NMR, and radiocarbon datasets that SPE-DOM composition remains largely conserved during WSDW formation. Moreover, the observed values for the process-specific I_BIO_ and I_PHOTO_ indices are typical of the deep-sea but are not frequently found in surface waters^37^. Photodegradation is potentially low in the Weddell Sea due to its high latitude and extensive ice cover. The absence of signals from intense biological production might be related to the notably long residence time of up to 12 years for water on the Weddell Sea continental shelf^24^. It is unusually high compared to other major sites of deep-water formation, such as the Ross Sea and the North Atlantic around Greenland, where shelf residence times are typically 3.5 years or less^25,26^. This extended residence time may allow for the complete remineralization of newly produced organic matter from phytoplankton in the Weddell Sea. In line with this explanation, the Ross Sea continental shelf accumulates substantial DOM, yet this extra carbon does not persist in AABW formed there^27^. Our observations indicate that the dominant, recalcitrant DOM signature in WSDW, which ultimately incorporates into AABW, was established before the mixing of dense shelf waters with relatively warm off-shelf waters.

### Conserved DOM properties in WSDW in a changing ocean

Deep and bottom water formation in the Weddell Sea has dramatically reduced over past decades^1,22^. It is plausible to assume that this slowdown in AABW formation could be due to climate change^22^, in particular since climate model simulations project a substantial further decline of AABW formation in the coming decades due to surface warming, sea ice decline, and enhanced ice shelf melt. A decline in deep and bottom water formation would also affect the amount and composition of DOM that is exported from the Weddell Sea to the deep ocean, with potentially long-term effects on carbon storage in the world ocean. However, here we show that the dissolved organic concentration, apparent ^14^C_SPE-DOC_ ages, molecular formulas (FT-ICR-MS) and structural properties (^1^H-NMR) of the SPE-DOM were conserved during WSDW formation (Figs. 2, 3). Furthermore, by studying SPE-DOM directly at the remote deep-water formation sites in the Weddell Sea (Fig. 1), we provide evidence that SPE-DOM in the main dense and deep-water masses was unaffected by shelf processes such as biological production or photochemical alterations (Figs. 2−4). DOM produced on the shelf was effectively removed, likely due to rapid remineralization of labile DOM to inorganic carbon^33^, enhanced by long residence times in the deep and large Weddell Sea shelf^24^. As warming of the Antarctic and Southern Ocean regions reduces sea ice formation^23^, CO_2_ exchange with the atmosphere and primary production could potentially increase^10,23,50^. This change could lead to greater production of fresh, labile DOM that, if not fully converted to inorganic DIC, might be detectable at a molecular level in the future.

The Southern Ocean is a strong carbon sink of approximately 1 PgC per year, representing around 40% of the global ocean uptake of anthropogenic CO_2_ emissions^51^. Considering the high variability over time and in the different regions of the Southern Ocean, as well as the differences in methodological approaches, estimates of this carbon uptake in the literature range between 0.5–2.0 PgC per year^51,52^. A recent study indicates that under ice-free conditions, a decrease of 0.14 PgC per year should be considered in those budgets^53^. Regarding dissolved organics in the open ocean, some models suggested that the downward flux of DOC could be significant in the Southern Ocean in the top 500 m of the water column^54^. Our observations in 2016 and 2021 did not align with this possibility, as there was no significant difference in DOC concentration across any of the seven water masses involved in WSDW formation, ranging from the surface to up to 4433 m depth (Welch-ANOVA; Fig. 2a; Tables S2–S4). Although our observations suggested negligible DOC transport among the water masses contributing to WSDW formation (Fig. 2a), substantial gross carbon export still occurs during deep-water formation. With production rates of newly-formed AABW of 8.4 Sv^55^, with 54% from dense shelf waters (4.5 Sv^55^;) and 46% of entrained WDW (3.9 Sv^55^;), and considering the average DOC concentrations per water mass that we measured (Dense shelf water DOC = 47.7 ± 6.6 µM; Off-shelf DOC = 50.2 ± 10.3 µM; Table S2; Data S1), the organic carbon transfer from the Weddell Sea to the abyssal ocean would be 0.155 ± 0.03 PgC per year (see calculations in Methods). These estimates are around ten times larger than the calculated flux of terrestrially-derived DOC from rivers into the deep waters of the Arctic Ocean, estimated as 0.0173 PgC per year^56^. Ocean warming and sea ice decline are expected to reduce the production of dense shelf waters and slow the formation of AABW^22^. As a result, the flux of DOC exported to the abyssal ocean will likely decline in the future. Our findings highlight DOM as a stable component of the regional Southern Ocean carbon cycle, as both DOC concentrations and DOM molecular composition remained highly similar during the formation of WSDW, without relevant transformations when upwelling WDW was transformed into dense shelf waters (Figs. 2−4). We conclude that in future climate projections with a potential increase in productivity in surface waters^10^, or an increased presence of mWDW on the southern Weddell Sea shelf^23^, the molecular signature of the dissolved fraction of organic carbon exported from the Weddell Sea into the abyssal ocean is unlikely to be affected.

## Methods

### Water masses classification

A major fraction of the dense-water masses forms on the southern Weddell Sea shelf^23^. There, water mass transformation proceeds via a series of distinguishable water masses (Fig. 1b, c). Heat exchange with the atmosphere leads to cooling and the formation of cold (<−1.6 °C) Winter Water (WW). Further, the intensive sea ice production leads to brine rejection and the formation of High Salinity Shelf Water (HSSW). During circulation beneath the large Filchner-Ronne ice shelf, HSSW cools below the surface freezing point (−1.9 °C) and slightly freshens by basal melt. This process ultimately forms Ice Shelf Water (ISW) which, together with HSSW, gets exported from the shelf as Dense Shelf Water overflow (DSWo) through topographic depressions such as the Filchner Trough^57–60^. During subduction of these dense shelf waters down the continental slope, they mix with circulating Warm Deep Water (WDW) from the Weddell Sea Gyre to form Weddell Sea Deep Water (WSDW), which eventually leaves the Weddell Gyre and contributes to AABW. At the shelf break, the so-called Antarctic Slope Front separates the cold shelf waters from the warmer water masses of the Weddell Gyre. Occasionally, cold WW and WDW (at +1 °C) mix to form modified Warm Deep Water (mWDW) with densities that allow on-shelf transport. Thus, the import of mWDW roughly balances the export of ISW^57–60^.

Based on conservative temperature, absolute salinity and potential density (σ_0_), following previous water mass definitions^57–60^, we grouped 116 samples into the following water masses: Winter Water (WW), Warm Deep Water (WDW), modified Warm Deep Water (mWDW), Ice Shelf Water (ISW), High Salinity Shelf Water (HSSW), Dense Shelf Water overflow (DSWo) and Weddell Sea Deep Water (WSDW) (Fig. 1b, c; Table S1). Detailed definitions in Table S1 were partly converted from potential temperature and in situ or absolute salinity from^57–60^. Furthermore, based on latitude, longitude, and maximum depth of the stations, we separated the different stations into these regions of the Weddell Sea: basin, peninsula outflow, inner shelf, slope Filchner-Trough (FT), sill, slope before FT, and shelf before FT (Fig. 2, S1). Considering the different regions (Fig. S1), eleven samples were discarded from the water mass categories WDW, WW, and mWDW to focus only on samples potentially mixing with ISW in the southern Weddell Sea shelf, and not the surroundings. They included one WDW sample located in the peninsula outflow, six WW samples from the shelf before FT, two mWDW samples from the basin, and two mWDW samples from the slope before FT. These eleven samples, together with the rest that were not categorized inside any of the seven water masses, were included as “other” (*n* = 62, gray, Fig. S2). Although they were discarded from the seven water masses included in deep-water formation, the “other” samples were also part of the statistical analyses (e.g., Fig. 3a), and none of those samples showed a specific pattern in their geochemical or molecular composition (Fig. 3a, S3; Data S1, S2).

### Sampling and onboard measurements

All samples were collected onboard the research icebreaker Polarstern during the cruises PS96 (06.12.2015 to 14.02.2016^29^;) and PS124 (03.02.2021 to 30.03.2021^30^;) to the southern Weddell Sea shelf^24^. All water samples (2L volume each) were obtained using a water sampling rosette (SeaBird SBE911plus) combined with a conductivity-temperature-depth (CTD) sensor and equipped with an additional sensor for oxygen (SBE43-1; SeaBird Electronics). A high-precision thermometer (SBE35, sn77/sw6345) was mounted on the rosette, and salinity was measured with the Optimare Precision Salinometer for post-cruise calibration of the conductivity sensors^30^. A total of 178 water column samples from 38 different stations in different regions of the Weddell Sea were subsampled for DOM (Data S1). The CTD sampled stations were located in the Weddell Sea, between 64−77°S and 11−52°W (Fig. 1a). Immediately after sampling, water samples were filtered (0.7 µm, Ø 47 mm, Whatman), preserved in the dark, frozen (−20 °C), and transported to the laboratories in Germany.

### Geochemical, isotopic and structural analyses

After unfreezing, the samples were acidified to pH 2 and extracted according to^19^ using solid-phase extraction (SPE) on styrene-divinylbenzene polymer-filled cartridges (1 g, Agilent Bond Elut PPL, USA). Dissolved organic carbon (DOC) and total dissolved nitrogen (TDN) concentrations were determined in triplicate from the filtered and acidified water samples by high-temperature catalytic oxidation on a Shimadzu TOC-VCPH analyzer. For the determination of carbon in the solid-phase extracts (SPE-DOC), aliquots of the methanol extracts were evaporated to dryness and redissolved in ultrapure water of pH 2. The accuracy of the DOC measurements was tested in each run against deep Atlantic seawater reference material provided by the consensus reference material project (Hansell Biogeochemistry Lab, University of Miami, USA). The overall extraction efficiencies were 34% in both PS96 and PS124 cruises. From a total of 178 water samples, after removing contaminated samples, we had a dataset of 106 DOC and 152 SPE-DOC samples (Data S1). From the SPE-DOC fraction, we analyzed the DOM ^14^C_SPE-DOC_ radiocarbon age (Fig. 2a; Table S3; Data S1), and the molecular composition at the structural level by ^1^H-NMR (Fig. 4).

The ^14^C_SPE-DOC_ radiocarbon dating analysis was performed on 54 SPE-DOC samples from PS124 following the protocol described for SPE-DOC dating in^61^. Extract volumes containing ca. 80 µg C were transferred into 50 µL liquid tin capsules (ELEMENTAR, S03 951 619) kept in a custom-made aluminum tray. The tray was placed on a hot plate at 40 °C until samples were dry. To ensure complete removal of MeOH, the samples were kept in a desiccator under mild vacuum at 60 °C for a minimum of 48 h. Radiocarbon analysis were carried out with the EA-GIS AMS setup at the AWI MICADAS following established standard operation procedures^62^. Radiocarbon data were normalized and blank-corrected against standard CO_2_ (produced from NIST Oxalic Acid II, NIST SRM4990C) and ^14^C-free CO_2_. Further, the tin capsule blank was determined and corrected for following the protocol described by ref. ^63^.

The ^1^H-NMR spectra were acquired on a Bruker Avance Neo 800 MHz instrument equipped with a 1.7 mm TCI microcryoprobe. Methanol from the solid-phase extracted aliquot was evaporated to dryness under a stream of nitrogen gas. The SPE-DOM was redissolved in 50 µL of CD_3_OD and transferred to 1.7 mm NMR tubes using a 100 µL Hamilton syringe. NMR experiments were performed at 298 K. The ^1^H-NMR spectra were recorded using the “noesygppr1d” pulse sequence. The number of time-domain points was set to 64 K, with a spectral width of 16 kHz. The acquisition time was two seconds, followed by a four-second relaxation delay. A 10 ms NOESY mixing time was used. The number of dummy scans and acquisition scans was set to 8 and 800, respectively, resulting in a total experiment time of ca. one hour. NMR spectra acquisition and processing were performed using TopSpin 4.4.1 (Bruker, Germany). Binning of the ^1^D−^1^H-NMR spectra was carried out using NMRProcFlow^64^. The bin resolution was 0.01 ppm, with a noise threshold of three. Water and methanol regions were excluded from the analysis. Prior to multivariate statistical analysis, the NMR data were sum-normalized. Bray-Curtis dissimilarity analysis (see Statistical analyses) showed instrumental variability of 0.4% over a 24 h period.

Additionally, we included in the discussion the calculation regarding the DOC transfer from the Weddell Sea to the deep ocean. Total DOC transfer from the Weddell Sea into the deep ocean was estimated as 0.155 ± 0.03 PgC per year. Calculation details were as follows:

- AABW production rate = 8.4 Sv ^55^ = 8.4 × 10^6^ m^3^ s^−1^, from which 4.5 Sv originates in DSW (F_DSW_) and 3.9 Sv comes from entrained warm water (F_WDW_).
- Fraction DSW (F_DSW_): DOC-DSW_average_ (HSSW, ISW, DSWo) = 47.7 ± 6.6 µM = 0.0477 mol m^−3^; F_DSW_ = 4.5 × 10^6^ x 0.0477 = 2.15 ×10^5 ^mol s^−1^.
- Fraction WDW (F_WDW_): DOC-WDW_average_ = 50.2 ± 10.3 µM = 0.0502 mol m^−3^; F_WDW_ = 3.9 × 10^6^ x 0.0502 = 1.96 × 10^5 ^mol s^−1^.
- Total DOC export in AABW: F_TOTAL_ = 2.15 × 10^5^ + 1.96 × 10^5^ = 4.11 10^5^ mol s^−1^ = 4.93 × 10^6 ^g s^−1^ = 1.55 × 10^14 ^g yr^−1^ = 0.155 PgC yr^−1^.
- The uncertainty was calculated from the DOC standard deviations: DOC-DSW = ± 6.6 µM = 13.8%; DOC-WDW = ± 10.3 µM = 20.5%; Average = ca. 17% = 0.03 PgC yr^−1^.
- Total DOC export from Weddell Sea to AABW = 0.155 ± 0.03 PgC yr^−1^.

### Molecular DOM characterization by FT-ICR-MS

All FT-ICR-MS samples from both cruises were analyzed together at the FT-ICR-MS of the University of Oldenburg (Germany). Mass spectra were obtained in negative ionization mode using a SolariX FT-ICR-MS with a 15 T magnet system (Bruker Daltonics) combined with an ESI source (Bruker Apollo II) with a needle voltage set to −4 kV. All samples were analyzed in a 1:1 volumetric mixture of methanol and ultrapure water. The concentration was adjusted to 2.5 mg C L^−1^. The samples were directly infused into the ESI source at a flow rate of 120 µL h^−1^. Mass spectrometry quality and reproducibility were monitored by analyzing twice a day an internal deep sea DOM reference sample (North Equatorial Pacific Intermediate Water; NEqPIW^65^). Two hundred transient scans in broadband mode were accumulated for each run, covering the mass range of 150−2000 Da. The spectra were calibrated as described in ref. ^66^. Internal calibration of the spectra was performed using the Bruker Daltonics Data Analysis software package with the help of an established calibration list for marine DOM, consisting of more than 100 mass calibration points of known molecular formulas. After calibration, the mass error was <0.1 ppm. Then, the mass lists were processed using ICBM-OCEAN^67^. ICBM-OCEAN uses a method detection limit (MDL) to account for noise and reduce systematic error, allowing for a more precise formula attribution. The MDL calculation is described in detail in^66,67^. Molecular formulas with C_1-100_, H_1-250_, O_1-100_, N_0-4_, S_0-2_ or P_0-1_ were assigned to peaks based on ICBM-OCEAN premises considering isotope ratio verifications, large homologous series network, and the smallest differences to the reference mass^67^. Only those masses assigned to molecular formulas were included in the analysis, and isotopologues were removed from further consideration. All samples from both cruises were analyzed in duplicates in alternative days and the mass spectra reported here are the mean of the replicate measurements. After quality controls, four samples from PS124 were removed from further analyses (“NA”, Data S2). Blank samples (ultrapure water at pH 2) were analyzed, and all of the DOM formulas detected in the blanks were combined into one sample, treated as contaminants and therefore removed from the final dataset (“Blank”, Data S2). The detected masses were normalized to the sum of all mass intensities of the corresponding sample that were used to calculate intensity-weighted averages of molar ratios (H/C, O/C, N/C, S/C, P/C, N/O, S/O; Table S2). The normalized signal intensities were used to calculate two indices: the Double Bond Equivalent (DBE) to assess the degree of unsaturation, and the modified aromaticity index (AI_mod_ > 0.5^68^) to calculate the proportion of aromatic and condensed aromatic compounds in total DOM, taking into consideration the abundance of carboxyl groups in natural organic matter (Table S2; Fig. S3). Three additional indexes were used for calculating the degree of degradation of the molecules (I_DEG_^36^), the molecular signature of biological DOM transformations (I_BIO_^37^), and the impact of photochemical alterations on the DOM (I_PHOTO_^37^). To assess the degree of saturation and oxidation, we classified all molecular formulas into eight chemically robust definitions^67^: (1) Aromatics _O-poor_ (AImod > 0.5 and O/C ≤ 0.5), (2) Aromatics _O-rich_ (AI_mod_ > 0.5 and O/C > 0.5), (3) Highly unsaturated _O-poor_ (AI_mod_ <0.5, H/C < 1.5 and O/C ≤ 0.5), (4) Highly unsaturated _O-rich_ (AImod <0.5, H/C < 1.5 and O/C > 0.5), (5) Unsaturated _O-poor_ (1.5 ≤ H/C ≤ 2 and O/C ≤ 0.5), (6) Unsaturated _O-rich_ (H/C ≥ 1.5, H/C ≤ 2 and O/C > 0.5), (7) Saturated _O-poor_ (H/C > 2 and O/C ≤ 0.5), (8) Saturated _O-rich_ (H/C > 2 and O/C > 0.5) (Fig. S3; Data S2). The proportion of each compound group is reported as the percentage of molecular formulas of a given compound group relative to the total number of molecular formulas detected in the samples (e.g., ref. ^39^; Fig. S3). This categorization offers a useful overview of likely molecular structures, though other structural possibilities may exist (e.g., ref. ^41^).

### Statistical analyses

Statistical analyses included correlations (Pearson; Table S4), analyses of variance (Welch-ANOVA; Table S3), and multivariate methods (Principal Coordinate Analysis; PCoA) based on Bray-Curtis dissimilarities Figs. 3, S2). Pearson linear correlations were calculated among all physicochemical, geochemical, and isotopic parameters (Pearson; R package “stats”, function “cor.test”; R Core Team, 2023; Table S4). Analysis of variance was calculated between WDW, WW, mWDW, HSSW, ISW, DSWo, and WSDW geochemical and molecular properties (Table S3). Welch-ANOVA was selected after considering the parametric data with unequal variances between groups, including a p-adjustment after Holm to prevent false positives and a Games-Howell post-hoc test to determine significantly different groups (Welch-ANOVA, R package “ggstatsplot”, function “ggbetweenstats”^69^; Table S3). The FT-ICR-MS dataset was analyzed by multivariate methods including a PCoA based on Bray-Curtis dissimilarities of the normalized signal intensities of each molecular formula (Bray-Curtis dissimilarity; R software package “vegan”, functions “vegdist”, “enfvit”^70^; R package “gplots”, function “heatmap.2”; R package “stats”, function “cmdscale”^70^; Fig. 3, S2). All the NEqPIW samples (*n* = 22) had, on average 9 ± 3% dissimilarity, with a minimum of 4% and a maximum of 17% dissimilarity. Still, Bray-Curtis dissimilarities were identified in our dataset, as we reached a maximum of 43% dissimilarity when comparing Weddell Sea samples within our dataset (Fig. S2). Results are displayed as a PCoA two-dimensional matrix with molecular parameters in arrows explaining the observed dissimilarities (Fig. 3a) and a heatmap with a dendrogram based on Bray-Curtis dissimilarities in percentage, with 0% dissimilarity being the same (Figs. 3b, 4c; refs. ^39,40^).

### Reporting summary

Further information on research design is available in the Nature Portfolio Reporting Summary linked to this article.

## Supplementary information

Supplementary Information

Supplementary Dataset 1

Supplementary Dataset 2

Reporting Summary

Transparent Peer Review file

## Data Availability

Supplementary Information Data file includes extended Methods, Supplementary Tables (S1–S4), and Supplementary Figs. (S1–S3). The FT-ICR-MS, physicochemical, geochemical and isotopic data have been deposited in Zenodo (https://doi.org/10.5281/zenodo.22768142).
