# Supplementary material for: Conserved molecular properties of dissolved organic matter during Weddell Sea Deep Water formation
Source: Nat Commun. 2026 Nov 10;17:11455. doi: 10.1038/s41467-026-79040-4 (PMC13751623; doi:10.1038/s41467-026-79040-4)
Supplement: Supplementary file 1 [file 41467_2026_79040_MOESM1_ESM.pdf]

Supplementary Information

Conserved molecular properties of dissolved organic matter during Weddell Sea Deep Water formation

Gonzalo V. Gomez-Saez*[1,2,3,4], Sarah K. Bercovici[5,6], Marit Renken[3], Sahithya P. B. Vemulapalli[5], Andreas Rogge[1,7], Hendrik Grotheer[1,2], F. Alexander Haumann[1,3], Moritz Holtappels+[1,2], Thorsten Dittmar+[5,8,9]

1) Alfred Wegener Institute Helmholtz Centre for Polar and Marine Sciences (AWI), Bremerhaven, Germany
2) MARUM Center for Marine Environmental Sciences, University of Bremen, Bremen, Germany
3) Ludwig-Maximilians-Universität (LMU) München, Munich, Germany
4) GeoBio-Center[LMU], Ludwig-Maximilians-Universität (LMU) München, Munich, Germany
5) Institute for Chemistry and Biology of the Marine Environment (ICBM), School of Mathematics and Science, Carl-von-Ossietzky Universität Oldenburg, Oldenburg, Germany
6) Marine Biogeochemistry, National Oceanography Centre, Southampton, UK
7) Institute of Environmental Physics, University of Bremen, Bremen, Germany
8) Helmholtz Institute for Functional Marine Biodiversity (HIFMB), University of Oldenburg, Oldenburg, Germany
9) Max Planck Institute for Marine Microbiology (MPI), Bremen, Germany

*) Corresponding author: g.gomez@lmu.de
+) These authors jointly supervised this work.

Methods (extended)

Water masses classification

A major fraction of the dense water masses forms on the southern Weddell Sea shelf [1]. There, water mass transformation proceeds via a series of distinguishable water masses. Heat exchange with the atmosphere leads to cooling and the formation of cold (< -1.6°C) Winter Water (WW). Further, the intensive sea-ice production leads to brine rejection and the formation of High Salinity Shelf Water (HSSW). During circulation beneath the large Filchner-Ronne ice shelf, HSSW cools below surface freezing point (-1.9°C) and slightly freshens by basal melt. This process ultimately forms Ice Shelf Water (ISW), which, together with HSSW, gets exported from the shelf through topographic depressions such as the Filchner Trough [2-5]. During subduction of these dense shelf waters down the continental slope, they mix with circulating Warm Deep Water (WDW) from the Weddell Sea gyre to form Weddell Sea Deep Water (WSDW), which eventually leaves the Weddell Gyre and contributes to AABW and global MOC. At the shelf break, the so-called Antarctic Slope Front separates the cold shelf waters from the warmer water masses of the Weddell Gyre. Occasionally, cold WW and WDW (at +1 °C) mix to form modified Warm Deep Water (mWDW) with densities that allow on-shelf transport. Thus, the import of mWDW roughly balances the export of DSW [2-5].

Based on conservative temperature, absolute salinity and potential density (σ_0), following [2-5], we grouped 116 samples into the following water masses: Winter Water (WW), Warm Deep Water (WDW), modified Warm Deep Water (mWDW), Ice Shelf Water (ISW), High Salinity Shelf Water (HSSW), Dense Shelf Water overflow (DSWo) and Weddell Sea Deep Water (WSDW) (Fig. 1B, 1C; Table S1). Detailed definitions in Table S1 were partly converted from potential temperature and in situ or absolute salinity from [2-5]. Furthermore, based on latitude, longitude and maximum depth of the stations, we separated the different stations by these regions of the Weddell Sea: Basin, Peninsula outflow, Inner shelf, Slope Filchner-Trough (FT), Sill, Slope before FT and Shelf before FT (Fig. 2, S1). Considering the different regions (Fig. S1), 11 samples were discarded from the water masses categories WDW, WW and mWDW, to focus only on samples potentially mixing with ISW in the southern Weddell Sea shelf, and not the surroundings. This included one WDW located in the peninsula outflow, six WW samples from the shelf before FT, two mWDW samples from the basin and two mWDW samples from the slope before FT. These 11 discarded samples together with the rest that were not categorized inside any of the seven water masses, were included as “other” (n=62, gray, Fig. S2). Although they were discarded from the seven water masses included in dense-water formation, the “other” samples were also included in the statistical analyses and none of those samples showed some specific pattern in its geochemical or molecular composition (Fig. S2; Data S1, S2).

Sampling and onboard measurements

All samples were collected onboard the icebreaker Polarstern during the cruises PS96 (06.12.2015 to 14.02.2016; [6]) and PS124 (03.02.2021 to 30.03.2021; [7]) to the southern Weddell Sea shelf [8]. All water samples (2L volume each) were obtained using a water sampling rosette (SeaBird SBE911plus) combined with a conductivity-temperature-depth (CTD) sensor and equipped with an additional sensor for oxygen (SBE43-1; SeaBird Electronics). A high precision thermometer (SBE35, sn77/sw6345) was

mounted on the rosette and salinity was measured with the Optimare Precision Salinometer for post-cruise calibration of the conductivity sensors [7]. A total of 178 water column samples from 38 different stations in different regions of the Weddell Sea were subsampled for DOM (Data S1). The CTD sampled stations were located in the Weddell Sea, between 64 - 77°S and 11 - 52°W (Fig. 1A). Immediately after sampling, water samples were filtered (0.7 µm, Ø 47 mm, Whatman), preserved in the dark, frozen (-20 °C), and transported to the laboratories in Germany.

Geochemical, isotopic and structural analyses

After unfreezing, the samples were acidified to pH 2, and extracted according to [9] using solid phase extraction (SPE) on styrene divinyl benzene polymer filled cartridges (1g, Agilent Bond Elut PPL, USA). Dissolved organic carbon (DOC) and total dissolved nitrogen (TDN) concentrations were determined in triplicates from the filtered and acidified water samples by high-temperature catalytic oxidation on a Shimadzu TOC-VCPH analyzer. For the determination of carbon in the solid-phase extracts (SPE-DOC), aliquots of the methanol extracts were evaporated to dryness and redissolved in ultrapure water of pH 2. The accuracy of the DOC measurements was tested in each run against deep Atlantic seawater reference material provided by the consensus reference material project (Hansell Biogeochemistry Lab, University of Miami, USA). The overall extraction efficiencies were 34% in both PS96 and PS124 cruises. From a total of 178 water samples, after removing contaminated samples, we had a dataset of 106 DOC and 152 SPE-DOC samples (Data S1). From the SPE-DOC fraction, we analyzed the DOM $^{14}C_{SPE\text{-}DOC}$ radiocarbon age (Fig. 2A; Table S3; Data S1), and the molecular composition on structural level by ^{1}H-NMR (Fig. 4).

The $^{14}C_{SPE\text{-}DOC}$ radiocarbon dating analysis was performed on 54 SPE-DOC samples from PS124 following the protocol described for SPE-DOC dating in [10]. Extract volumes containing ca. 80 µgC were transferred into 50 µL liquid tin capsules (ELEMENTAR, S03 951 619) kept in a custom-made aluminum tray. The tray was placed on a hot plate at 40 °C until samples were dry. To ensure complete removal of MeOH the samples were kept in a desiccator under mild vacuum at 60 °C for a minimum of 48 h. Radiocarbon analyses were carried out with the EA-GIS AMS setup at the AWI MICADAS following established standard operation procedures [11]. Radiocarbon data were normalized and blank corrected against standard CO_2 (produced from NIST Oxalic Acid II, NIST SRM4990C) and ^{14}C-free CO_2. Further, the tin capsule blank was determined and corrected for following the protocol described by [12].

The ^{1}H-NMR spectra were acquired on a Bruker Avance Neo 800 MHz instrument equipped with a 1.7 mm TCI microcryoprobe. Methanol from the solid-phase extracted aliquot was evaporated to dryness under a stream of nitrogen gas. The SPE-DOM was redissolved in 50 µL of CD_3OD and transferred to 1.7 mm NMR tubes using a 100 µL Hamilton syringe. NMR experiments were performed at 298 K. The ^{1}H-NMR spectra were recorded using the “noesygppr1d” pulse sequence. The number of time-domain points was set to 64 K, with a spectral width of 16 kHz. The acquisition time was two seconds, followed by a four seconds relaxation delay. A 10 ms noesy mixing time was used. The number of dummy scans and acquisition scans as set to 8 and 800, respectively, resulting in a total experiment time of ca. one hour. NMR spectra acquisition and processing were performed using TopSpin 4.4.1 (Bruker, Germany). Binning of the ^{1}D-^{1}H-NMR spectra was carried out using NMRProcFlow [13]. The bin resolution was 0.01 ppm, with a noise threshold of three. Water and methanol regions

were excluded from the analysis. Prior to multivariate statistical analysis, the NMR data were sum-normalized. Bray-Curtis dissimilarity analysis (see below Statistical analyses) showed instrumental variability of 0.4% over a 24-h period.

Additionally, we included in the discussion the calculation regarding the DOC transfer from the Weddell Sea to the deep ocean. Total DOC transfer from the Weddell Sea into the deep ocean was estimated as 0.155 ± 0.03 PgC per year. Calculation details were as follows:

- AABW production rate = 8.4 Sv [14] = 8.4 x 10^6 m^3 s^{-1}, from which 4.5 Sv originates in DSW (F_{DSW}) and 3.9 Sv comes from entrained warm water (F_{WDW}).
- Fraction DSW (F_{DSW}): DOC-$DSW_{average}$ (HSSW, ISW, DSWo) = 47.7 ± 6.6 µM = 0.0477 mol m^{-3}; F_{DSW} = 4.5 x 10^6 x 0.0477 = 2.15 x 10^5 mol s^{-1}.
- Fraction WDW (F_{WDW}): DOC-$WDW_{average}$ = 50.2 ± 10.3 µM = 0.0502 mol m^{-3}; F_{WDW} = 3.9 x 10^6 x 0.0502 = 1.96 x 10^5 mol s^{-1}.
- Total DOC export in AABW: F_{TOTAL} = 2.15 x 10^5 + 1.96 x 10^5 = 4.11 10^5 mol s^{-1} = 4.93 x 10^6 g s^{-1} = 1.55 x 10^{14} g yr^{-1} = 0.155 PgC yr^{-1}.
- The uncertainty was calculated from the DOC standard deviations: DOC-DSW = ± 6.6 µM = 13.8%; DOC-WDW = ± 10.3 µM = 20.5%; Average = ca. 17% = 0.03 PgC yr^{-1}.
- Total DOC export from Weddell Sea to AABW = 0.155 ± 0.03 PgC yr^{-1}.

Molecular DOM characterization by FT-ICR-MS

All FT-ICR-MS samples from both cruises were analyzed together at the FT-ICR-MS of the University of Oldenburg (Germany). Mass spectra were obtained in negative ionization mode using a SolariX FT-ICR-MS with a 15T magnet system (Bruker Daltonics) combined with an ESI source (Bruker Apollo II) with a needle voltage set to -4 kV. All samples were analyzed in a 1:1 volumetric mixture of methanol and ultrapure water. The concentration was adjusted to 2.5 mg C L^{-1}. The samples were directly infused into the ESI source at a flow rate of 120 µL h^{-1}. Mass spectrometry quality and reproducibility was monitored by analyzing twice a day an internal deep sea DOM reference sample (North Equatorial Pacific Intermediate Water; NEqPIW, [15]). Two hundred transient scans in broadband mode were accumulated for each run, covering the mass range of 150 - 2000 Da. The spectra were calibrated as described in [16]. Internal calibration of the spectra was performed using the Bruker Daltonics Data Analysis software package with the help of an established calibration list for marine DOM, consisting of more than 100 mass calibration points of known molecular formulas. After calibration, the mass error was <0.1 ppm. Then, the mass lists were processed using ICBM-OCEAN [17]. ICBM-OCEAN uses a method detection limit (MDL) to account for noise and reduce systematic error, allowing for a more precise formula attribution. The MDL calculation is described in detail in [16,17]. Molecular formulas with C_{1-100}, H_{1-250}, O_{1-100}, N_{0-4}, S_{0-2} or P_{0-1} were assigned to peaks based on ICBM-OCEAN premises considering isotope ratio verifications, large homologous series network, and the smallest differences to the reference mass [17]. Only those masses assigned to molecular formulas were included in the analysis and isotopologues were removed from further considerations. All samples from both cruises were analyzed in duplicate in alternative days and the mass spectra reported here are the mean of the replicate measurements. After quality controls, four samples from PS124 were removed from further analyses ("NA", Data S2). Blank samples (ultrapure water at pH 2) were analyzed and all of the DOM formulas detected in the blanks were combined into one

sample, treated as contaminants and therefore removed from the final dataset ("Blank", Data S2). The detected masses were normalized to the sum of all mass intensities of the corresponding sample that were used to calculate intensity-weighted averages of molar ratios (H/C, O/C, N/C, S/C, P/C, N/O, S/O; Table S2). The normalized signal intensities were used to calculate two indices: the Double Bond Equivalent (DBE) to assess the degree of unsaturation, and the modified aromaticity index ($AI_{mod} > 0.5$; [18]) to calculate the proportion of aromatic and condensed aromatic compounds in total DOM taking into consideration the abundance of carboxyl groups in natural organic matter (Table S1; Fig. S3). Three additional indexes were used for calculating the degree of degradation of the molecules (I_{DEG} [19]), the molecular signature of biological DOM transformations (I_{BIO} [20]) and the impact of photochemical alterations into the DOM (I_{PHOTO} [20]). To assess the degree of saturation and oxidation, we classified all molecular formulas into eight chemically robust definitions [17]: (1) Aromatics $_{O\text{-poor}}$ (AImod > 0.5 and O/C ≤ 0.5), (2) Aromatics $_{O\text{-rich}}$ (AI_{mod} > 0.5 and O/C > 0.5), (3) Highly unsaturated $_{O\text{-poor}}$ (AI_{mod} < 0.5, H/C < 1.5 and O/C ≤ 0.5), (4) Highly unsaturated $_{O\text{-rich}}$ (AImod < 0.5, H/C < 1.5 and O/C > 0.5), (5) Unsaturated $_{O\text{-poor}}$ (1.5 ≤ H/C ≤ 2 and O/C ≤ 0.5), (6) Unsaturated $_{O\text{-rich}}$ (H/C ≥ 1.5, H/C ≤ 2 and O/C > 0.5), (7) Saturated $_{O\text{-poor}}$ (H/C > 2 and O/C ≤ 0.5), (8) Saturated $_{O\text{-rich}}$ (H/C > 2 and O/C > 0.5) (Fig. S3; Data S2). The proportion of each compound group is reported as the percentage of molecular formulas of a given compound group relative to the total number of molecular formulas detected in the samples (e.g. [21]; Fig. S3). This categorization offers a useful overview of likely molecular structures, though other structural possibilities may exist (e.g. [22]).

Statistical analyses

Statistical analyses included correlations (Pearson; Table S4), variance analyses (Welch-ANOVA; Table S3) and multivariate methods (PCoA based on Bray-Curtis dissimilarities; Fig. 3, S2). Pearson linear correlations were calculated among all physicochemical, geochemical and isotopic parameters (Pearson; R package "stats", function "cor.test"; R Core Team, 2023; Table S4). Analysis of variance was calculated between WDW, WW, mWDW, HSSW, ISW, DSWo and WSDW geochemical and molecular properties (Table S3). Welch-ANOVA was selected after considering the parametric data with unequal variances between groups, including a p-adjustment after Holm to prevent false positives and Games-Howell post-hoc test to determine significantly different groups (Welch-ANOVA, R package "ggstatsplot", function "ggbetweenstats" [23]; Table S3). The FT-ICR-MS dataset was analyzed by multivariate methods including a Principal Coordinate Analysis (PCoA) based on Bray-Curtis dissimilarities of the normalized signal intensities of each molecular formula (Bray-Curtis dissimilarity; R software package "vegan", functions "vegdist", "enfvit" [24]; R package "gplots", function "heatmap.2"; R package "stats", function "cmdscale"; [24]; Fig. 3, S2). Results are displayed as a PCoA two-dimensional matrix with molecular parameters in arrows explaining the observed dissimilarities in the PCoA (Fig. 3B) and a heatmap with a dendrogram based on Bray-Curtis molecular dissimilarities in percentage, with 0% dissimilar being the same (Fig. 3B, 4C; e.g. [21,25]). All the NEqPIW samples (n=22) had on average 9 ± 3% of dissimilarities with a minimum of 4% and a maximum of 17% dissimilarity. Still, Bray-Curtis dissimilarities were identified in our dataset as we reached a maximum of 43% dissimilarity when comparing Weddell Sea samples within our dataset (Fig. S2). All figures in the manuscript were generated using R and edited with Affinity Designer 2. The map in Fig. 1A was generated using "rnaturalearth", "ggplot2", "sf" and "ggspatial" libraries.

Table S1: Water mass definitions modified from the literature[2-5].
Definitions were partly converted from potential temperature, and in situ or absolute salinity from the literature. Shown conversions are referenced to the center of the study area on the southern Weddell Sea shelf (-75 °N, -35 °E) and surface pressure (p0) while conversions were applied dynamically in the study. sFP abbreviates surface freezing point. max. $pden_{WDW\_slope}$ is the maximum potential density of waters at the continental slope stations which were shallower than the maximum depth of the Filchner Trough sill. Waters with potential densities > $pden_{WDW\_slope}$ are thus able to subduct down the continental slope. For visualization see also Fig 1.

**Water mass**	**Abbreviation**	**Abs. salinity [g kg^{-1}]**	**Con. temperature [°C]**	**Pot. density anomaly [kg m^{-3}]**
**Winter Water**	WW	34.466 - 34.617	>sFP <-1.696	
**Warm Deep Water**	WDW	34.808 - 34.888	>0.2016	
**Modified Warm Deep Water**	mWDW	34.57 - 34.808	-1.696 - 0.2016	<27.7957 (max. $pden_{WDW\_slope}$ –0.01)
**Ice Shelf Water**	ISW	34.57 – 34.8211	< sFP	
**High Salinity Shelf Water**	HSSW	>34.8211	<-1.697	
**Dense Shelf Water overflow**	DSWo	<34.8211	sFP – -0.7961	>max. $pden_{WDW\_slope}$
**Weddell Sea Deep Water**	WSDW		-0.7961 – 0.2019	>max. $pden_{WDW\_slope}$

Table S2: Averages and standard deviations of physicochemical, geochemical, isotopic and molecular parameters.

	Water mass	Warm Deep Water	Winter Water	modified Warm Deep Water	High Salinity Shelf Water	Ice Shelf Water	Dense Shelf Water overflow	Weddell Sea Deep Water
	Abbreviation	WDW	WW	mWDW	HSSW	ISW	DSWo	WSDW
	n	11	30	13	13	23	10	16
Physicochemical, geochemical and isotopic parameters	Salinity (absolute, g/kg)	34.850 ± 0.007	34.525 ± 0.032	34.664 ± 0.050	34.883 ± 0.011	34.783 ± 0.029	34.777 ± 0.027	34.831 ± 0.012
	T (conservative, °C)	0.565 ± 0.238	-1.799 ± 0.040	-1.010 ± 0.546	-1.948 ± 0.032	-1.992 ± 0.048	-1.652 ± 0.322	-0.240 ± 0.260
	Depth (m)	923 ± 398	132 ± 81	384 ± 108	554 ± 189	478 ± 85	784 ± 735	2222 ± 1036
	O_2 (µM)	205.8 ± 8.7	323.4 ± 6.6	277.0 ± 25.9	316.8 ± 1.3	319.7 ± 3.4	304.0 ± 16.3	230.0 ± 10.9
	DOC (µM)	50 ± 10 (n=6)	54 ± 6 (n=15)	54 ± 6 (n=6)	47 ± 4 (n=7)	47 ± 7 (n=15)	50 ± 8 (n=6)	52 ± 10 (n=12)
	TDN (µM)	36 ± 1	33 ± 1	34 ± 1	33 ± 1	33 ± 1	34 ± 1	35 ± 2
	SPE-DOC (µM)	21 ± 2 (n=8)	20 ± 2 (n=30)	20 ± 3 (n=12)	20 ± 1 (n=11)	19 ± 3 (n=15)	18 ± 2 (n=8)	20 ± 2 (n=11)
	F14C	0.4859 ± 0.0197	0.4941 ± 0.0196	0.4969 ± 0.0193	0.4960 ± 0.0191	0.4972 ± 0.0209	0.4871	0.4866 ± 0.0196
	Δ^{14}C-age (‰)	-521 ± 10	-510 ± 17	-506 ± 14	-507 ± 23	-495 ± 24	-517	-533 ± 19
	^{14}C-age (years)	5841 ± 175 (n=8)	5665 ± 283 (n=14)	5598 ± 230 (n=7)	5622 ± 369 (n=6)	5420 ± 372 (n=7)	5778 (n=1)	6050 ± 336 (n=11)
Molecular parameters (FT-ICR-MS)	# formulas	3338 ± 793	3162 ± 816	3014 ± 441	3762 ± 1060	3829 ± 1065	3368 ± 787	3485 ± 674
	m/z	419 ± 12	411 ± 12	414 ± 9	411 ± 17	418 ± 13	413 ± 12	417 ± 13
	H/C	1.27 ± 0.02	1.27 ± 0.02	1.27 ± 0.01	1.27 ± 0.02	1.27 ± 0.01	1.27 ± 0.01	1.27 ± 0.02
	O/C	0.46 ± 0.02	0.46 ± 0.01	0.46 ± 0.01	0.45 ± 0.02	0.46 ± 0.01	0.45 ± 0.01	0.46 ± 0.02
	N/C	0.019 ± 0.001	0.020 ± 0.002	0.019 ± 0.001	0.021 ± 0.002	0.020 ± 0.002	0.020 ± 0.002	0.019 ± 0.001
	S/C	0.001 ± 0.001	0.001 ± 0.001	0.001 ± 0.000	0.002 ± 0.001	0.002 ± 0.001	0.001 ± 0.001	0.001 ± 0.001
	P/C	0.002 ± 0.001	0.002 ± 0.001	0.002 ± 0.001	0.003 ± 0.002	0.003 ± 0.001	0.002 ± 0.001	0.002 ± 0.001
	N/O	0.049 ± 0.005	0.052 ± 0.006	0.049 ± 0.003	0.056 ± 0.009	0.055 ± 0.007	0.053 ± 0.006	0.051 ± 0.006
	S/O	0.004 ± 0.002	0.004 ± 0.003	0.003 ± 0.002	0.005 ± 0.003	0.005 ± 0.003	0.004 ± 0.002	0.004 ± 0.003

DBE	8.5 ± 0.3	8.4 ± 0.3	8.4 ± 0.2	8.4 ± 0.4	8.5 ± 0.3	8.4 ± 0.2	8.5 ± 0.3
AI_{mod}	0.24 ± 0.00	0.24 ± 0.00	0.24 ± 0.00	0.24 ± 0.01	0.24 ± 0.01	0.24 ± 0.00	0.24 ± 0.01
I_{PHOTO}	0.20 ± 0.02	0.20 ± 0.02	0.19 ± 0.02	0.22 ± 0.05	0.21 ± 0.02	0.21 ± 0.01	0.21 ± 0.04
I_{BIO}	0.19 ± 0.02	0.21 ± 0.02	0.20 ± 0.02	0.22 ± 0.05	0.20 ± 0.02	0.20 ± 0.02	0.20 ± 0.03
I_{DEG}	0.73 ± 0.05	0.69 ± 0.05	0.71 ± 0.03	0.69 ± 0.07	0.72 ± 0.04	0.71 ± 0.04	0.72 ± 0.04
Aromatics, %	1.78 ± 0.78	1.97 ± 0.80	1.63 ± 0.50	2.52 ± 1.08	2.46 ± 0.71	2.14 ± 0.55	2.00 ± 0.97
Highly Unsat., %	91.24 ± 2.89	90.17 ± 2.94	90.84 ± 2.15	89.11 ± 4.13	89.70 ± 2.05	89.94 ± 1.72	90.67 ± 3.16
Unsaturated, %	6.49 ± 1.92	7.28 ± 1.91	7.05 ± 1.42	7.70 ± 2.63	7.23 ± 1.30	7.36 ± 1.25	6.83 ± 1.95
Saturated, %	0.14 ± 0.09	0.18 ± 0.14	0.11 ± 0.08	0.23 ± 0.18	0.20 ± 0.10	0.18 ± 0.10	0.16 ± 0.13

Table S3: Welch-ANOVA test comparing the variance of WDW, WW, mWDW, HSSW, ISW, DSWo and WSDW water masses.
The only parameters not included were ^{14}C-age (as one of the water masses (DSWo) contained only one value), and salinity and temperature, as they were used to categorize the water masses.

	**Welch-Anova test $CI_{95\%}$ (0.00 – 1.00)**			
	F_{Welch}	p	$\omega^2 p$	n
Depth (m)	(7, 44.76) = 53.10	1.37 e-19	0.87	178
O_2 (µM)	(7, 47.78) = 377.44	1.46 e-39	0.98	178
DOC (µM)	(7, 25.73) = 2.27	0.06	0.21	106
TDN (µM)	(7, 50) = 23.45	9.50 e-14	0.73	178
SPE-DOC (µM)	(7, 35.78) = 1.52	0.19	0.08	152
m/z	(7, 46.53) = 1.74	0.12	0.09	178
C, #	(7, 46.26) = 2.26	0.05	0.14	178
H, #	(7, 45.29) = 2.20	0.05	0.14	178
O, #	(7, 46.55) = 1.42	0.22	0.05	178
N, #	(7, 48.97) = 2.63	0.02	0.17	178
S, #	(7, 47.95) = 1.86	0.10	0.10	178
P, #	(7, 46.82) = 0.48	0.84	0.00	178
H/C	(7, 46.14) = 0.83	0.57	0.00	178
O/C	(7, 46.43) = 1.84	0.10	0.10	178
N/C	(7, 49.75) = 3.95	1.66 e-03	0.26	178
S/C	(7, 47.72) = 2.28	0.04	0.14	178
P/C	(7, 46.68) = 0.43	0.88	0.00	178
N/O	(7, 48.21) = 2.94	0.01	0.19	178
S/O	(7, 47.19) = 1.66	0.14	0.08	178
DBE	(7, 46.80) = 1.75	0.12	0.09	178
AI_{mod}	(7, 46.37) = 1.29	0.28	0.04	178
NOSC	(7, 46.46) = 1.40	0.23	0.05	178
Aromatics %	(7, 47.32) = 2.79	0.02	0.18	178
Highly Unsaturated, %	(7, 46.76) = 0.94	0.48	0.00	178
Unsaturated, %	(7, 46.40) = 0.76	0.63	0.00	178
Saturated, %	(7, 48.73) = 1.48	0.20	0.06	178

Table S4: Pearson correlation coefficient ("*r*") of physicochemical, geochemical and isotopic parameters in all samples (n=178).
Significant correlations are marked with (*) meaning a p-value < 0.05.

Pearson´s correlation	Salinity (g/kg)	T (°C)	Depth (m)	O_2 (µM)	^{14}C- age (years)	DOC (µM)	TDN (µM)	SPE-DOC (µM)
Salinity (g/kg)	1.00							
T (°C)	0.30*	1.00						
Depth (m)	0.51*	0.50*	1.00					
O_2 (µM)	-0.65*	-0.85*	-0.66*	1.00				
^{14}C-age (years)	0.40*	0.43*	0.45*	-0.51*	1.00			
DOC (µM)	-0.20*	0.02	-0.07	0.03	-0.15	1.00		
TDN (µM)	0.70*	0.34*	0.39*	-0.59*	0.45*	-0.30*	1.00	
SPE-DOC (µM)	-0.05	0.14	0.13	-0.15	-0.01	0.50*	-0.16*	1.00

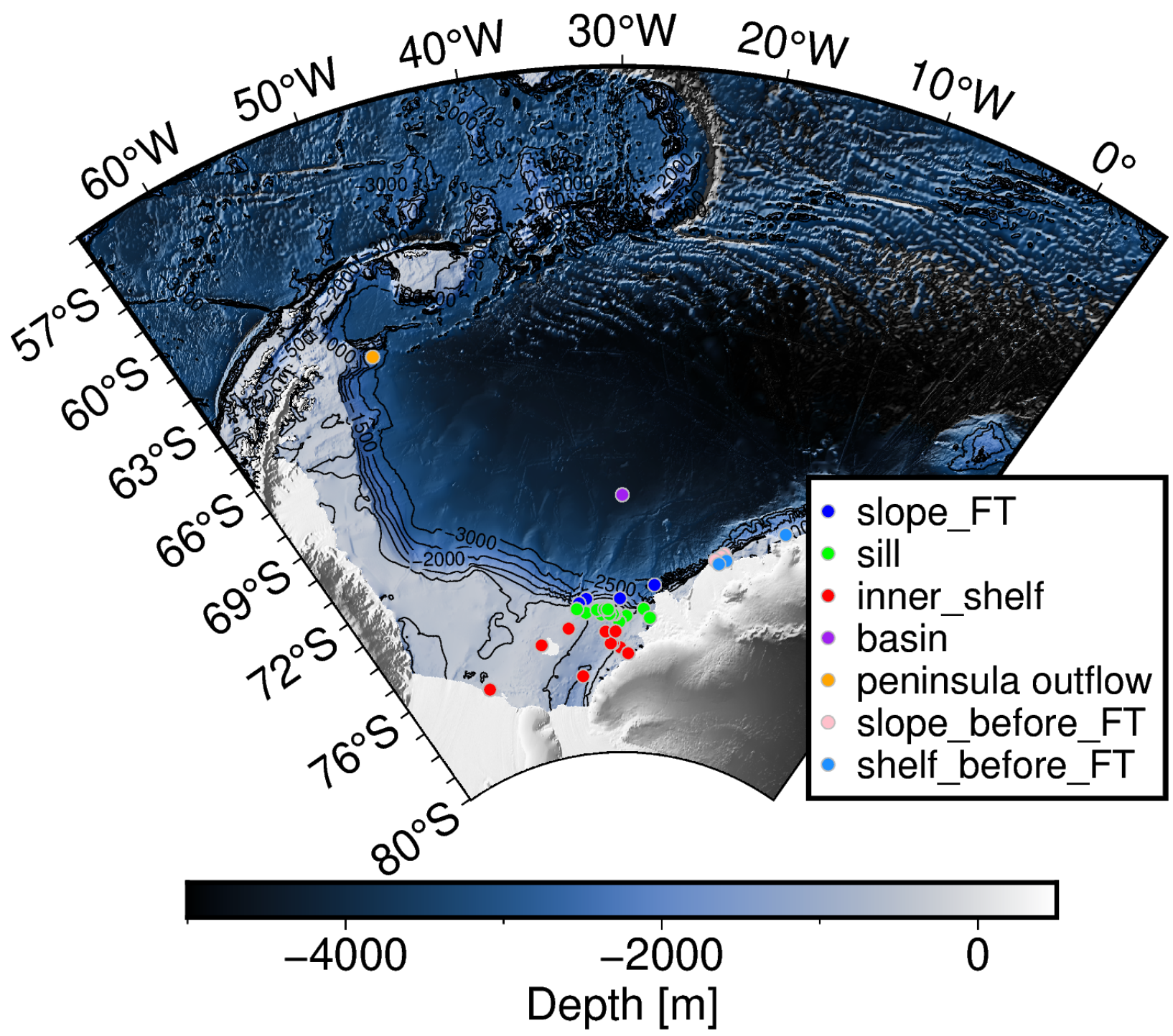

Region	Latitude (°)	Longitude (°)	Maximum depth (m)
**Basin**	> -74.85		> 3500
**Peninsula outflow**	> -70	< -40	
**Inner Shelf**	<= -75.2		
**Slope Filchner-Trough (FT)**	> -74.85	< -25	> 1000<br>< 3500
**Sill**	<= -74.57<br>> 75.2	< -25	< 1000
**Slope before FT**	> -74.85	> -25	> 1000<br>< 3500
**Shelf before FT**	> -74.85	> -25	< 1000

Fig. S1: Regional, areal characterization of the different stations at the Weddell Sea.

Based on latitude, longitude and maximum depth of the stations, we separated the different stations by region of the Weddell Sea (Fig. S3) into “slope Filchner-Trough (FT)” (dark blue), “sill” (green), “inner shelf” (red), “basin” (purple), “peninsula outflow” (orange), “slope before FT” (pink) and “shelf before FT” (light blue). Detailed categorization is explained in the Methods.

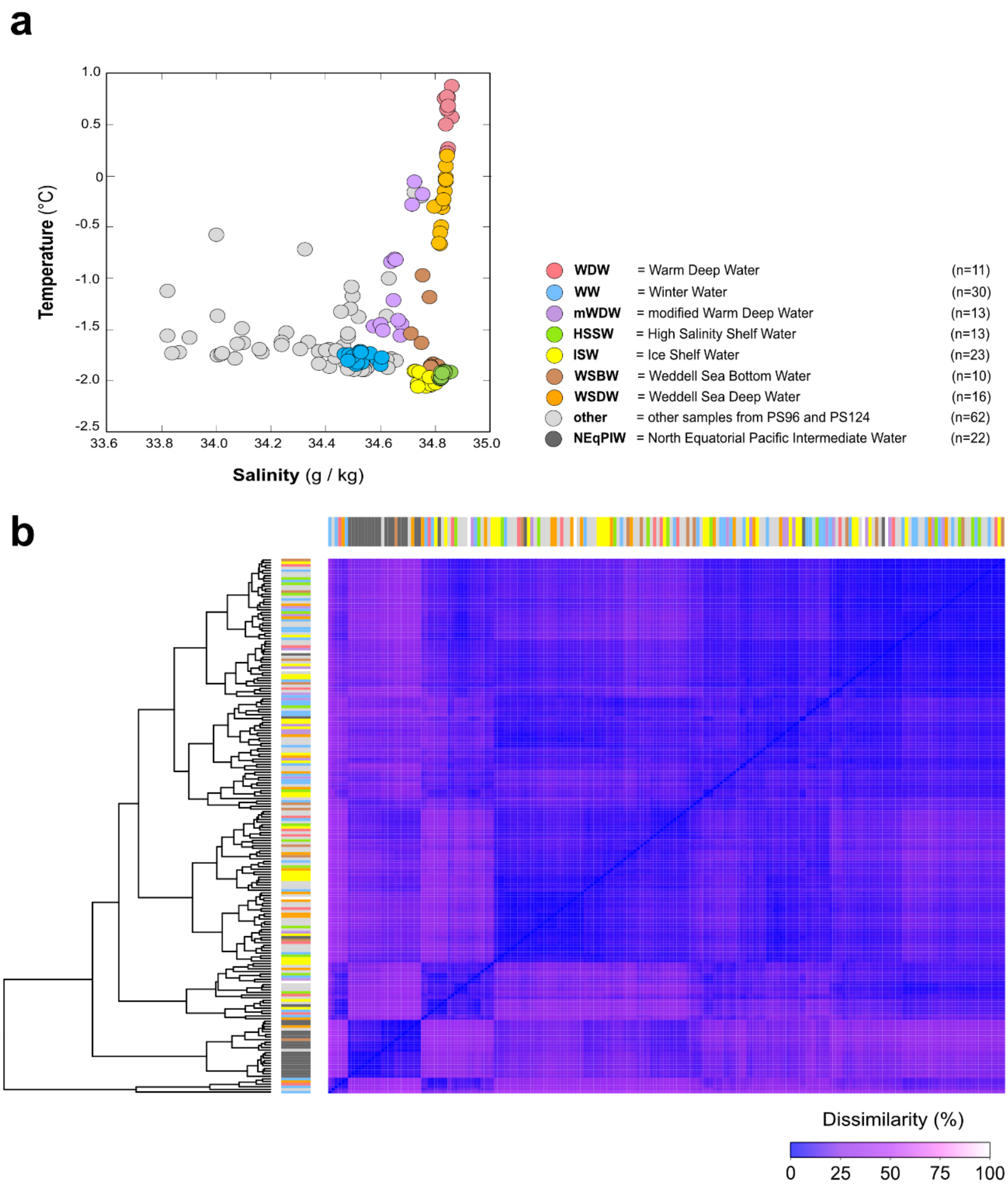

Fig. S2: Water samples and Bray-Curtis molecular dissimilarity analysis including all samples (n=178) obtained from PS96 and PS124.

The analysis considers the relative signal intensities of molecular formulas detected by FT-ICR-MS, with white representing 100% dissimilar (totally different or not present at all) and blue representing 0% dissimilar (the same). Samples are ordered by dissimilarity. Same analysis for the seven water masses directly connected to Weddell Sea deep water formation is presented in Fig. 3B (but with scale of 0 – 50% dissimiliarity).

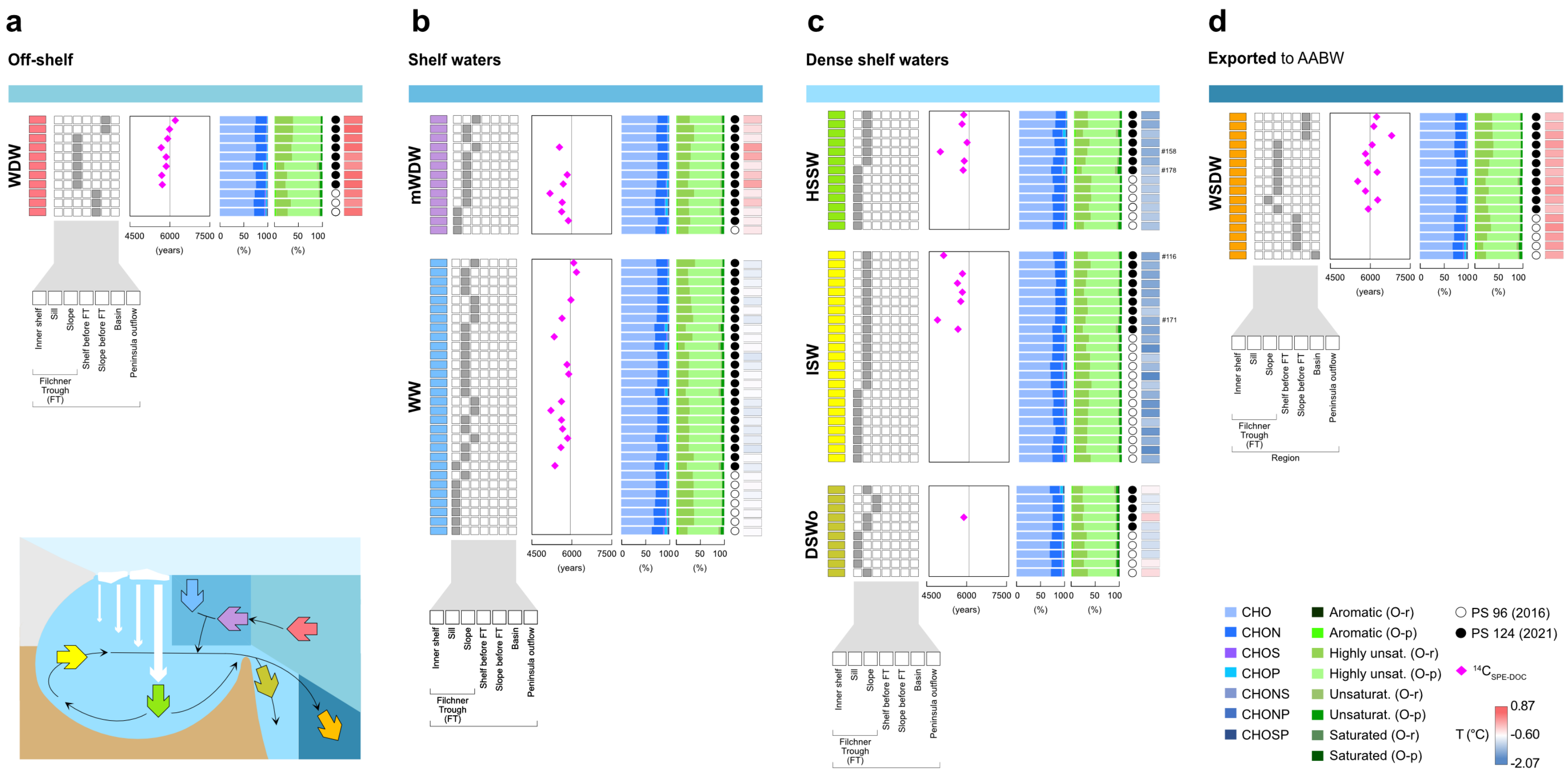

Fig. S3: Highly similar DOM composition and radiocarbon $^{14}C_{SPE\text{-}DOC}$ age across Weddell Sea water masses involved in deep water formation.

Individual composition of all samples categorized into the seven different water masses involved in WSDW formation. A: Off-shelf, warm waters: WDW. B: Shelf waters: WW, mWDW. C: Dense shelf waters: HSSW, ISW, DSWo. D: Exported to AABW: WSDW. From left to right, it is visualized the: Areal distribution within the Filchner-Trough (FT): inner shelf, sill, slope, or around FT: shelf before FT, slope before FT, basin and peninsula outflow; Apparent radiocarbon ^{14}C-age (pink); Relative abundance of each DOM sample containing different molecular formulas (left, CHO compounds, with or without N, S and/or P) or each compound category (right, Aromatics, Highly unsaturated, Unsaturated or Saturated); Expedition: PS96 (2016, white) or PS124 (2021, black); and Temperature: from blue (minimum = -2.07°C) to red (maximum = +0.87°C). The four samples within the shelf waters which presented the highest molecular differences (#178) and lowest ^{14}C-age (#158, #116, #171) are labelled in panel C

References (Supplementary Information)

1 Nissen, C., Timmermann, R., Hoppema, M., Gürses, Ö. & Hauck, J. Abruptly attenuated carbon sequestration with Weddell Sea dense waters by 2100. *Nat. Commun.* **13**, 3402 (2022).

2 Carmack, E. C. & Foster, T. D. Circulation and distribution of oceanographic properties near the Filchner Ice Shelf. *Deep Sea Res. Oceanogr. Abstr.* **22**, 77-90 (1975).

3 Grosfeld, K. & Gerdes, R. Circulation beneath the Filchner Ice Shelf, Antarctica, and its sensitivity to changes in the oceanic environment: a case-study. *Ann. Glaciol.* 27, 99-104 (1998).

4 Foldvik, A. et al. Ice shelf water overflow and bottom water formation in the southern Weddell Sea. *J. Geophys. Res. Oceans* **109**, C02015 (2004).

5 Nicholls, K. W., Østerhus, S., Makinson, K., Gammelsrød, T. & Fahrbach, E. Ice-ocean processes over the continental shelf of the southern Weddell Sea, Antarctica: A review. *Rev. Geophys.* **47**, RG3003 (2009).

6 Schröder, M. The expedition PS96 of the research vessel *POLARSTERN* to the southern Weddell Sea in 2015/2016. *Ber. Polar- Meeresforsch.* **703**, 142 pp (2016).

7 Hellmer, H. H. & Holtappels, M. The expedition PS124 of the Research Vessel *POLARSTERN* to the southern Weddell Sea in 2021. *Ber. Polar- Meeresforsch.* **755**, 237 pp (2021).

8 Janout, M. A. et al. FRIS Revisited in 2018: On the circulation and water masses at the Filchner and Ronne ice shelves in the southern Weddell Sea. *J. Geophys. Res. Oceans* **126**, e2021JC017269 (2021).

9 Dittmar, T., Koch, B., Hertkorn, N. & Kattner, G. A simple and efficient method for the solid-phase extraction of dissolved organic matter (SPE-DOM) from seawater. *Limnol. Oceanogr. Methods* **6**, 230-235 (2008).

10 Hildebrand, T. et al. Transformation of dissolved organic matter by two Indo-Pacific sponges. *Limnol. Oceanogr.* **67**, 2483-2496 (2022).

11 Mollenhauer, G., Grotheer, H., Gentz, T., Bonk, E. & Hefter, J. Standard operation procedures and performance of the MICADAS radiocarbon laboratory at Alfred Wegener Institute (AWI), Germany. *Nucl. Instrum. Methods Phys. Res. B* **496**, 45-51 (2021).

12 Sun, S. et al. 14C blank assessment in small-scale compound-specific radiocarbon analysis of lipid biomarkers and lignin phenols. *Radiocarbon* **62**, 207-218 (2020).

13 Jacob, D., Deborde, C., Lefebvre, M., Maucourt, M. & Moing, A. NMRProcFlow: a graphical and interactive tool dedicated to 1D spectra processing for NMR-based metabolomics. *Metabolomics* **13**, 36 (2017).

14 Akhoudas, C. H. et al. Ventilation of the abyss in the Atlantic sector of the Southern Ocean. *Sci. Rep.* **11**, 6760 (2021).

15 Green, N. W. et al. An intercomparison of three methods for the large-scale isolation of oceanic dissolved organic matter. *Mar. Chem.* **161**, 14-19 (2014).

16 Riedel, T. & Dittmar, T. A method detection limit for the analysis of natural organic matter via Fourier transform ion cyclotron resonance mass spectrometry. *Anal. Chem.* **86**, 8376-8382 (2014).

17 Merder, J. et al. ICBM-OCEAN: Processing ultrahigh-resolution mass spectrometry data of complex molecular mixtures. *Anal. Chem.* **92**, 6832-6838 (2020).

18 Koch, B. P. & Dittmar, T. From mass to structure: an aromaticity index for high-resolution mass data of natural organic matter. *Rapid Commun. Mass Spectrom.* **20**, 926-932 (2006).

19 Flerus, R. et al. A molecular perspective on the ageing of marine dissolved organic matter. *Biogeosciences* **9**, 1935-1955 (2012).

20 Bercovici, S. K., Wiemers, M., Dittmar, T. & Niggemann, J. Disentangling biological transformations and photodegradation processes from marine dissolved organic matter composition in the global ocean. *Environ. Sci. Technol.* **57**, 21145-21155 (2023).

21 Gomez-Saez, G. V. et al. Sulfurization of dissolved organic matter in the anoxic water column of the Black Sea. *Sci. Adv.* **7**, eabf6199 (2021).

22 Vemulapalli, S. P. B., Griesinger, C. & Dittmar, T. Expanding the limits of structural characterization of marine dissolved organic matter using nonuniform sampling frequency-reversed edited HSQC NMR. *Anal. Chem.* **95**, 14770-14776 (2023).

23 Patil, I. Visualizations with statistical details: The "ggstatsplot" approach. J. *Open Source Softw.* **6**, 3167 (2021).

24 Oksanen, J. et al. vegan: Community Ecology Package. *R package version 2.6-4* https://CRAN.R-project.org/package=vegan (2022).

25 Gomez-Saez, G. V., Pohlabeln, A. M., Stubbins, A., Marsay, C. M. & Dittmar, T. Photochemical alteration of dissolved organic sulfur from sulfidic porewater. *Environ. Sci. Technol.* **51**, 14144-14154 (2017).
