# Supplementary material for: Conserved molecular properties of dissolved organic matter during Weddell Sea Deep Water formation
Source: Nat Commun. 2026 Nov 10;17:11455. doi: 10.1038/s41467-026-79040-4 (PMC13751623; doi:10.1038/s41467-026-79040-4)
Supplement: Supplementary file 3 [file 41467_2026_79040_MOESM5_ESM.pdf]

nature portfolio

Peer Review File

Conserved molecular properties of dissolved organic matter during Weddell Sea Deep Water formation

Corresponding Author: Dr Gonzalo Gomez-Saez

This file contains all reviewer reports in order by version, followed by all author rebuttals in order by version.

Version 0:

Reviewer comments:

Reviewer #1

(Remarks to the Author)
Review of Dissolved organic carbon export to the abyssal ocean unaffected by Weddell Sea Deep Water formation
The authors extracted portions of DOM by SPE, and analyzed variation of C-14, FTICR-MS, and NMR signatures along the Weddell Sea Deep Water formation pathway. They found little changes and concluded that properties of DOM do not change during the AABW formation.
The findings are new and the science is robust. So I support publication of the manuscript in Nat Comm.
I would like the authors to include brief discussion on the following. They examined only SPE-DOM, which is suggested to be refractory and old. I think that most variation and changes in molecular composition along the pathway would occur in the labile and semi-labile fraction, i.e., the non-retained portion. This does not influence the findings presented in the manuscript. However, this is something that needs to be discussed clearly as caveat of their study.

Reviewer #2

(Remarks to the Author)
The paper makes the case that the export of dissolved organic carbon during deepwater formation in the Southern Ocean may be an important event in the abyssal circulation of dissolved organic matter (DOM) through the ocean. The authors infer that since the Southern Ocean, particularly the Weddell Sea, is experiencing significant changes to its biogeochemical (changes in the amount and type of primary production) and physical dynamics (timing and extent of ice melting, the rate of deep-water formation) this might impact the composition of DOM over time in the deep sea. The authors further imply that changes in the composition of DOM could results in changes to its reactivity and ultimately the amount of carbon stored in this reservoir.

To address this hypothesis the authors measured the spectrometric and isotopic characteristics of DOM collected by solid phase extraction (SPE). They find that there is little difference in spectrometric/isotopic properties between waters coming onto the Weddell Sea shelf and deeper waters exiting the shelf that contribute to the formation of Antarctic Bottom Water. The authors conclude that recent changes in Weddell Sea biogeochemistry and physical processes are unlikely to impact DOM composition or (by inference) reactivity/storage with that caveat that in the future there might be changes in DOM compositon.

The most puzzling aspect of the paper to me is why one would think that contemporary changes in bottom water formation would result in a significant change in DOM composition, particularly in the (radiocarbon old) solid phase extractable (SPE) fraction of DOM. The residence time of recalcitrant marine DOC is many thousands of years indicating several trips around the ocean. Each trip exposes DOM to changes in the biogeochemical and physical environment, but deep-sea DOM (and even surface DOM) is remarkably consistent in its spectrometric fingerprint and bulk chemical properties.
My quick, back of the envelope, calculations (which agree with the calculations made in this paper on page 6) show that the annual export of DOC via Weddell Sea deepwater is several orders of magnitude larger than TOTAL primary production across the Sea. Assuming that only a small fraction of total primary production is transformed into DOC, the difference between export and production is even larger. The consistency between DOC and DOC radiocarbon values in the water column support that, quantitatively, production in the basin does not affect export. Given all this, my assumption (null hypothesis) would be that biogeochemical processes in the basin do not significantly impact the molecular composition of

DOM. I therefore did not find the results of the paper noteworthy.

To my mind the authors seem to be taking what happens to DOC in temperate and tropical systems and implying this should be expected in the Southern Ocean. But the mixing regimes and residence times of these systems are very different as reflected in the large changes with depth in DOC concentration and radiocarbon values, and the modest changes with depth in DOC composition at temperate and tropical sites. I would expect that rapid mixing in the Weddell Sea quickly erases what little impact biogeochemical processes in the basin could have on DOC composition. The statement (page 5, Line 237) …"While the exact cause of the absence of signals from intense biological production in the deep-water masses is unknown" … is not a mystery to me. My expectation going into the study would be that there should be little or no change in DOM composition, which is indeed what was measured.

A second and related issue I had with the paper is that the results apply to < 50% of DOM and not the entire DOC reservoir. This is never made clear to the reader. SPE DOM in not representative of other fractions of DOM. DOM collected by ultrafiltration (~ 25-30% DOM) is primarily polysaccharides that are not captured by solid phase extraction. Likewise, there is a significant fraction of DOM (another ~25-30%) that is presumably low in molecular weight and not captured by solid phase extraction. The fact that this fraction is not efficiently captured by SPE suggests it is chemically distinct from SPE-DOM. One could argue that the composition of DOM is in fact changing, just not in the SPE fraction.

Much of the interpretation in the paper centers around the FT-ICR-MS data (Figs. 2&3). FT-ICR-MS data is inherently qualitative, and it is unknown what fraction of SPE-DOM this analysis tracks (for example the lower-than-expected capture of organic nitrogen by SPE cited by others), and how changes in ion intensity quantitatively relate to changes in the larger chemical composition and properties of SPE-DOM.

In summary, there are significant caveats & limitations to the conclusions of the paper (as exemplified in the title) that there is (or potentially will be) no change in DOM composition with changing deep-water formation. The "no observable" change in DOM composition is what I expected. Although I understand the motivation to have all results published in a high impact journal, I do not see this as a high impact, high citation paper, and feel it more properly belongs in an AGU or Frontiers journal.

Specific comments

Page 1
Title- I think the title not supported by the study, as only a fraction of DOM was characterized.
Line 29 "Therefore, carbon cycling processes, such as the production and turnover of dissolved organic matter (DOM), in AABW formation sites are crucial." Not clear what is being said. Crucial how? to what?
Line 32 That you are studying SPE DOM and that this is only a fraction of the total should be included in the abstract.

Page 2
Line 69-70 "The spatial overlap…suggests carbon export to greater depths". Not necessarily. It has been amply demonstrated that DOC concentration does not correlate with primary productivity. There is no reason to think productivity leads to export of DOC. Global sections of DOC concentration have no obvious component of export from the Southern Ocean into the deep Pacific. The uncertainty in the observational DOC data is too large to see a contribution if one exists.
Line 76 "Dense water formation….deep-sea reservoir". Not clear what is being said. All deep sea water and effectively all DOC originates from the surface. There may be small inputs of DOC from sediments in some places, but as far as we know these inputs are quantitatively negligible.
Line 82 "unrecognizable biomolecules". Readers may not know what you mean by this.
Line 82 "DOM persistence…known biochemical features". Again readers may not know what this sentence means, and it seems besides the point with regard to this paper.
Line 84 "…its great reservoir size and long…"
Line 84-96. It is not clear to me what the point of this paragraph is. I think it can be largely deleted.

Page 3
Line 146 "possible imprints on DOM pool size or reactivity". I do not understand what is being said here. Please clarify. Are you saying you are looking for changes in DOM composition as the concentration of DOC changes?
Lines 146- "For FT-ICR-MS and 1H-NMR…prior to extraction (Fig.2; Table S2)." This sentence should go at the beginning of the paragraph (line 126), before any statement of property conservation so that the reader knows boundary conditions of the observations. It is important to include a statement that SPE captures < 50% of DOC.
Line 147 "Solid-phase extraction (SPE) with modified styrene divinyl benzene polymer sorbents (PPL) is the most efficient extraction method to date". The relative efficiency of SPE is not this issue. The more important point is the amount of DOM SPE recovers (< 50% DOC). One could suggest that the fraction most likely to be impacted by biogeochemical processes, not the quantitatively most recoverable fraction, is the fraction of DOM to focus on here.
Line 158 "Molecular formula data provide insights into the degradation state of Marine DOM." I think the molecular degradation index developed by Fleurs (ref 37) does not provide any insight into DOM degradation. The radiocarbon old samples in the Fleurs paper show no differences in relative ion intensities. In this study the radiocarbon value between samples does not change (-510 per mil) and like the Fleurs paper, there is no change in molecular properties. But the issue of molecular degradation index aside, doesn't this section repeat what has already been said in the preceding text, that vigorous mixing in the Southern Ocean homogenizes DOM enough that there is no changes in composition between the samples? I am not sure what the point of this discussion is.

Page 4
line 190 “The typical decrease in DOM 14C-age in surface waters was absent in the Weddell Sea (Fig. 2A, 4A), indicating little to no net-input of modern carbon to DOM, despite the productive surface waters at the study site”. Are you making a distinction here between “radiocarbon” modern carbon and carbon “recently synthesized” by primary or secondary production? My recollection is that due to intense vertical mixing the radiocarbon value of DIC in the euphotic zone of the Southern Ocean is old (~ -260 per mil) – reflecting deep water values (I am thinking of the WOCE dataset). In that case “recently synthesized” DOC would not be “radiocarbon” modern, as it is in temperate and tropical waters where exchange with the atmosphere introduces bomb radiocarbon into the DIC, but instead have values of -200 to -300 per mil. If you are discussing “recently synthesized” carbon (and I think you are) then even a 10% contribution to DOM would fall within the 22 per mil uncertainty of the Δ14CSPE-DOC values, and could not be tracked with radiocarbon. This makes the subsequent discussion much less relevant.

Line 203. This is to be expected, no change in DOC concentration, rapid mixing, deep waters to near the surface.

Page 5
Lines 220-235. I don’t understand the point of this discussion.
Line 237 “While the exact cause of the absence of signals from intense biological production in the deep-water masses is unknown”. See my general comments. I don’t think the authors have made a case as to why we would expect there to be a significant difference in the molecular composition of DOM between surface and deep waters here.
Line 272 “DOM produced on the shelf was effectively removed likely due to rapid remineralization of labile DOM to inorganic carbon, enhanced by the long residence times of the deep and large Weddell Sea shelf 25. We suggest that the labile DOM produced at the surface became undetectable because it was rapidly transformed into refractory DOM 55, and this loss was not balanced by the production of new labile DOM in surface waters 11,30.”
I am unclear as to what is being said. The first sentence says that labile DOM is rapidly remineralized, while the second sentence says that labile DOM is undetectable because it is rapidly transformed into refractory DOM.

The technical aspects of the paper are fine. The sampling and analyses are all through established methods. The figures and captions are all well constructed and easy to follow.

Version 1:

Reviewer comments:

Reviewer #1

(Remarks to the Author)
The points raised by the first round of review appears to be incorporated in the revision. I support publication of the paper in Nature Communications.

Reviewer #2

(Remarks to the Author)
The authors have successfully revised the manuscript. I have some small additional comments below, some still related to my original review that the authors may have missed, and some points where I have no issue with what is being said, but where I think the text is not clear and could be improved.

Specific comments:

Lines 70-71. “mainly due….Ocean”. This should be deleted. It is not important to your point. There are major oceanographic expeditions to the Southern Ocean and Antarctic seas every year. For scientists in Europe, East Asia, and the US, access is not a problem. There are many reasons why scientists choose not to do this.

Lines 87-89, isn’t the point you are trying to make is that although DOC concentrations suggest that Weddell Sea processing may not quantitatively impact the amount of carbon exported, changes in composition could impact the quality of that organic matter export. If this is your point, then the authors might replace the last sentence of the paragraph with a better summary than a statement that the Southern Ocean has been undersampled. (see my comment line 103)

101 “In the Arctic Ocean…signature(32).” This sentence seems out of place. What does sea ice formation in the arctic have to do with this paragraph? The references cited studied the influence of sea ice formation on terrestrial organic matter, etc. which is quite different from the organic matter discussed here. This factoid and these studies are not strong support for the case you are trying to make. One can stretch and try to connect the two, but it is a weak argument easily rebutted. It does not help your paper.

103 “Nevertheless….abyss”. This sentence seems to belong at the end of the preceding paragraph (line 89) where you make the case that composition may change without a change in concentration. I suggested on lines 87-89 that you write a better summary statement. This might be it.

134 “the SPE-DOM analytical window”…

141 “taken together…broadly representative…deep ocean” as I indicated in my initial review, there is strong evidence that the SPE-DOM fraction is NOT representative of the broader DOM pool. My impression from the author’s response was that they accepted this view.

As I pointed out last time, the high molecular weight DOM pool which is about the same size as the SPE-DOM pool, has a different radiocarbon (by several hundred per mil or several thousand years), stable isotope, and molecular composition than SPE-DOM. How is SPE-DOM representative of the HMWDOM pool? This statement and the supporting evidence that preceded it should be deleted. The paragraph should continue from line 136 directly onto line 143 (“nitrogen content (36). In our study we focused…”

Line 147 “Molecular properties of SPE-DOM were conserved…”

Line 171 “Radiocarbon dating of SPE-DOM…”

Line 178 “the youngest D14C(SPE-DOC) values….” (you use the “older” terminology elsewhere in the paragraph/paper. Since isotope values are negative numbers, this is the highest not the lowest value….)

Line 192 “…D14C values…” add a space

Line 196-198. “This relatively small gradient reflects the upwelling of aged deep waters that supply an already old DOC pool to the Southern Ocean surface.”

This statement is true everywhere in the ocean, even where radiocarbon gradients are large. Upwelling of old DOC brings the apparent age of DOC in low latitude surface waters to about 2000 yrs. I suspect for most readers this will not be a clear explanation for the small gradient in radiocarbon. The small gradient is due the speed and intensity (depth) of the rapid vertical mixing of surface and deep water compared to low latitude regions (just as it does for nutrients, temperature, etc).

Line 199 “due to the addition of modern radiocarbon into labile DOM” (or something like that, to distinguish radiocarbon from new production, which do not necessarily track each other).

Line 199 “The typical decrease in DOM 14C age, combined with no significant change in DOM composition was absent from the Weddell Sea”. As written, this says there is a change in DOM composition with depth in the Weddell Sea (“no change” was absent). I don’t think you mean that.

The text between lines 199-201 should be revised. The sentence infers that there are compositional differences between surface and deep waters in the Southern Ocean that are absent from the Weddell Sea. While you show compositional differences are absent from the Weddell Sea, I don’t think you ever say or show explicitly that compositional differences with depth have been observed in the Southern Ocean.

Line 204 “likely supported by….from remineralization”. I don’t understand the basis for this statement. What are the authors saying? The citation (as I remember it, it is for an old paper) attributes changes in carbon export to mineral ballasting. I don’t understand the connection to remineralization as discussed here and carbon export by sinking particles.

Line 207 “modern” is ambiguous. Do you mean labile DOC?

Line 225 “associated with the upwelling of deep waters”. I think you mean with the rapid vertical mixing of surface and deep waters since upwelling occurs everywhere (see above).

Line 233 change “is related..” to “might be related..” since you do not really know why the signals are absent.

Line 242-254. This paragraph could be deleted. I am not sure what the point of the paragraph is. The point about the patterns of primary production in the Weddell Sea and how they interact with DOC accumulation is opaque to the reader as is the sentence about autotrophic and heterotrophic production. I did not understand what the authors were trying to convey.

Line 247 Since we do not know the environmental conditions of the Weddell Sea in detail, change “Under the specific environmental conditions..” to “Under some conditions microheterotrophs can outcompete…”. The citation only says that if there is enough labile substrate, then heterotrophs can compete with autotrophs for iron. This is true of other nutrients, if there is enough substrate, which is always the issue….

Line 258 “Our findings are in line with the most recent global oceanic DOC model suggesting rapid remineralization of labile DOC as a limiting factor controlling its contribution to long-term carbon storage.” This is saying that if DOM is rapidly remineralized it cannot contribute to long-term carbon storage. Isn’t that redundant?

My own view is that lines 256-262 could be deleted as they do not add to the discussion. The point of the paragraph is from lines 262-268.

Line 281 change DOM to SPE-DOM

Line 282 change DOM to SPE-DOM

Line 298 change “on” to “in”. …dissolved organics in the open ocean….

Open Access This Peer Review File is licensed under a Creative Commons Attribution 4.0 International License, which permits use, sharing, adaptation, distribution and reproduction in any medium or format, as long as you give appropriate credit to the original author(s) and the source, provide a link to the Creative Commons license, and indicate if changes were made.
In cases where reviewers are anonymous, credit should be given to 'Anonymous Referee' and the source.
The images or other third party material in this Peer Review File are included in the article’s Creative Commons license, unless indicated otherwise in a credit line to the material. If material is not included in the article’s Creative Commons license and your intended use is not permitted by statutory regulation or exceeds the permitted use, you will need to obtain permission directly from the copyright holder.
To view a copy of this license, visit https://creativecommons.org/licenses/by/4.0/

Reviewer #1

Review of Dissolved organic carbon export to the abyssal ocean unaffected by Weddell Sea Deep Water formation
The authors extracted portions of DOM by SPE, and analyzed variation of C-14, FTICR-MS, and NMR signatures along the Weddell Sea Deep Water formation pathway. They found little changes and concluded that properties of DOM do not change during the AABW formation.
The findings are new and the science is robust. So I support publication of the manuscript in Nat Comm.

Response: We would like to thank the Reviewer #1 for the positive comments on our manuscript. We believe that the input of both reviewers has significantly improved the quality of our resubmitted article.

I would like the authors to include brief discussion on the following. They examined only SPE-DOM, which is suggested to be refractory and old. I think that most variation and changes in molecular composition along the pathway would occur in the labile and semi-labile fraction, i.e., the non-retained portion. This does not influence the findings presented in the manuscript. However, this is something that needs to be discussed clearly as caveat of their study.

Response: Thank you for the suggestion. In the resubmitted version of our manuscript, we have clarified our focus on the SPE fraction in the abstract (L33-34, L37), introduction (L84-85, L104, L119) and results and discussion (L125, L127-145, L151, L213). In addition, we have included a new panel with SPE-DOM concentrations in Figure 2A. The newly added paragraph at the beginning of the Results & Discussion reads as follows (L127-145):

„Our findings consistently showed that SPE-DOM composition across Weddell Sea water masses involved in deep-water formation was highly similar, both at the molecular formula level (FT-ICR-MS; Fig. 2, 3) and in structural characteristics (^{1}H-NMR; Fig. 4). For FT-ICR-MS and ^{1}H-NMR, samples had to be concentrated and desalted. Solid-phase extraction (SPE) with modified styrene divinyl benzene polymer sorbents (PPL) is the most efficient extraction method to date [19,35]. Our SPE-DOC recovery (34%; Fig. 2A; Table S2) was within the range of previous SPE extraction efficiencies for Antarctic seawater DOC (14 – 42% [20,21]). Compared to total DOM, SPE-DOM analytical window selectively captures low molecular weight DOM [36] likely enriched in semi-labile and recalcitrant compounds [36,37], high degradation indices [21,37,38] and low organic nitrogen content [36]. Although some studies reported low $\Delta^{14}C_{SPE\text{-}DOC}$ values in SPE-DOM compared to total DOM [36,39,40], radiocarbon ages of bulk and SPE-extracted DOC were not significantly different in deep waters [36], and bulk DOC and SPE-DOC behaved conservatively during deep-water mixing [11]. Moreover, the ^{14}C ages of DOC in the Southern Ocean (approximately 5200 – 5800 ^{14}C years [41 42 43]) are in the same range as those reported here for SPE-DOM (Fig. 2A, 4A). Taken together, previous observations suggest that the PPL-extracted fraction is broadly representative of the larger DOC pool, especially in the deep ocean. In our study, we focused on this fraction of DOM for molecular, isotopic and radiocarbon characterization and combined the SPE-DOM with physicochemical and DOC measurements obtained prior to the extraction.“

Reviewer #2

The paper makes the case that the export of dissolved organic carbon during deep water formation in the Southern Ocean may be an important event in the abyssal circulation of dissolved organic matter (DOM) through the ocean. The authors infer that since the Southern Ocean, particularly the Weddell Sea, is experiencing significant changes to its biogeochemical (changes in the amount and type of primary production) and physical dynamics (timing and extent of ice melting, the rate of deep-water formation) this might impact the composition of DOM over time in the deep sea. The authors further imply that changes in the composition of DOM could results in changes to its reactivity and ultimately the amount of carbon stored in this reservoir.
To address this hypothesis the authors measured the spectrometric and isotopic characteristics of DOM collected by solid phase extraction (SPE). They find that there is little difference in spectrometric/isotopic properties between waters coming onto the Weddell Sea shelf and deeper waters exiting the shelf that contribute to the formation of Antarctic Bottom Water. The authors conclude that recent changes in Weddell Sea biogeochemistry and physical processes are unlikely to impact DOM composition or (by inference) reactivity/storage with that caveat that in the future there might be changes in DOM composition.

Response: We would like to thank the Reviewer #2 for the helpful comments on our manuscript. They have significantly improved the resubmitted version.

The most puzzling aspect of the paper to me is why one would think that contemporary changes in bottom water formation would result in a significant change in DOM composition, particularly in the (radiocarbon old) solid phase extractable (SPE) fraction of DOM. The residence time of recalcitrant marine DOC is many thousands of years indicating several trips around the ocean. Each trip exposes DOM to changes in the biogeochemical and physical environment, but deep-sea DOM (and even surface DOM) is remarkably consistent in its spectrometric fingerprint and bulk chemical properties.
My quick, back of the envelope, calculations (which agree with the calculations made in this paper on page 6) show that the annual export of DOC via Weddell Sea deep water is several orders of magnitude larger than TOTAL primary production across the Sea. Assuming that only a small fraction of total primary production is transformed into DOC, the difference between export and production is even larger. The consistency between DOC and DOC radiocarbon values in the water column support that, quantitatively, production in the basin does not affect export. Given all this, my assumption (null hypothesis) would be that biogeochemical processes in the basin do not significantly impact the molecular composition of DOM. I therefore did not find the results of the paper noteworthy.
To my mind the authors seem to be taking what happens to DOC in temperate and tropical systems and implying this should be expected in the Southern Ocean. But the mixing regimes and residence times of these systems are very different as reflected in the large changes with depth in DOC concentration and radiocarbon values, and the modest changes with depth in DOC composition at temperate and tropical sites. I would expect that rapid mixing in the Weddell Sea quickly erases what little impact biogeochemical processes in the basin could have on DOC composition. The statement (page 5, Line 237) …“While the exact cause of the absence of signals from

intense biological production in the deep-water masses is unknown" … is not a mystery to me. My expectation going into the study would be that there should be little or no change in DOM composition, which is indeed what was measured.

Response: We appreciate this important comment. We agree that conservation of SPE-DOM during deep-water formation may be anticipated from a mixing and residence-time perspective. However, there are also reasons to expect DOM modifications within Antarctic shelf systems, particularly in the highly productive Weddell Sea. Seasonal sea-ice melt and freshening stimulate intense phytoplankton blooms that produce labile DOM (Berg et al., 2020, *GRL*). Previous work in the Ross Sea showed that this recently produced DOC can persist within dense shelf waters over their 3–8 year residence time (Bercovici et al., 2017, *L&O*), demonstrating that shelf-derived organic matter can survive long enough to potentially influence newly formed bottom waters. In addition, newly formed AABW exhibits elevated DIC concentrations that have been attributed to remineralization of organic matter on the shelf (Zhang et al., 2023, *GBC*), indicating that biogeochemical processing does affect the properties of waters exported to the abyss. Therefore, while conservation of DOM is a reasonable null hypothesis, there are mechanistic reasons to test whether dense water formation alters DOM composition. This is now highlighted with new text in the introduction (L98-106).

Moreover, our study provides high-resolution molecular and isotopic observations from a region where such datasets are scarce (L67-71). This is relevant information for carbon budgets and representations of DOM export during AABW formation in global models. Our dataset was measured over two extensive sampling campaigns with multiple analytical approaches, allowing us to estimate DOC transfer from the Weddell Sea to the abyssal ocean (0.155 ± 0.03 PgC per year; L40, L309), representing around ten times more export of DOC than Arctic rivers (L312). While future climate projections indicate high variability across various factors in the Southern Ocean and AABW, our findings suggest that present-day changes in Weddell Sea deep-water formation are unlikely to substantially modify the molecular composition of the SPE-DOM exported to the abyss (L41-42; L320-321).

In addition, our study novelty relies on answering a gap in knowledge that is now highlighted in the resubmitted manuscript (L103-106): the unknown impact of dense water formation on the molecular properties of DOM. Specifically, our characterization aims to address the following questions: Are there molecular changes in the DOM exported to the abyssal ocean, even in the absence of a DOC increase in deep waters? How does dense water formation impact DOM composition? We agree with the reviewer that DOC can only be quantitatively relevant as an export mechanism if it accumulates (Carlson et al., 1998; *L&O*). This statement has now been included in our resubmitted manuscript (L78-79). However, the response of the DOM pool to surface ocean processes is not only expressed by the amount of DOC but also by its molecular composition (Hansell et al., 2026; Dittmar and Lennartz, 2026; L79-82). The revised manuscript now clarifies this distinction and provides contextual comparisons with sea-ice DOM dynamics in the Arctic Ocean (Jørgensen et al., 2015, *L&O*; Kong et al., 2026, *Nat Geosci*) (L101-102).

Considering this (and the specific comment suggesting a title change, see below), we have also adapted the title to focus on the conserved molecular properties of DOM during WSDW formation (L1-2) and added new text to the introduction (L98-106). To

better explain the global importance of our findings, the resubmitted manuscript now incorporates new text based on the following four references: Carlson et al., (1998): ref. #15; Jørgensen et al., (2015): ref. #31, Repeta and Aluwihare, (2026): ref. 35; von Berg et al., (2020): ref. #30.

A second and related issue I had with the paper is that the results apply to < 50% of DOM and not the entire DOC reservoir. This is never made clear to the reader. SPE DOM in not representative of other fractions of DOM. DOM collected by ultrafiltration (~ 25-30% DOM) is primarily polysaccharides that are not captured by solid phase extraction. Likewise, there is a significant fraction of DOM (another ~25-30%) that is presumably low in molecular weight and not captured by solid phase extraction. The fact that this fraction is not efficiently captured by SPE suggests it is chemically distinct from SPE-DOM. One could argue that the composition of DOM is in fact changing, just not in the SPE fraction.

Much of the interpretation in the paper centers around the FT-ICR-MS data (Figs. 2&3). FT-ICR-MS data is inherently qualitative, and it is unknown what fraction of SPE-DOM this analysis tracks (for example the lower-than-expected capture of organic nitrogen by SPE cited by others), and how changes in ion intensity quantitatively relate to changes in the larger chemical composition and properties of SPE-DOM.

In summary, there are significant caveats & limitations to the conclusions of the paper (as exemplified in the title) that there is (or potentially will be) no change in DOM composition with changing deep-water formation. The "no observable" change in DOM composition is what I expected. Although I understand the motivation to have all results published in a high impact journal, I do not see this as a high impact, high citation paper, and feel it more properly belongs in an AGU or Frontiers journal.

Response: In the resubmitted version of our manuscript, we have clarified our focus on the SPE fraction in the abstract (L33-34, L37), introduction (L84-85, L104, L119) and results and discussion (L125, L127-145, L151, L213). In addition, we have included a new panel with SPE-DOM concentrations in Figure 2A. As suggested by the reviewer, we have now included exact numbers on SPE recovery in marine samples (L84-85), and in our study compared to previous studies in the region (L132-133). In addition, we have included a new panel with SPE-DOM concentrations in Figure 2A. The newly added paragraph at the beginning of the Results & Discussion reads as follows (L127-145):

„Our findings consistently showed that SPE-DOM composition across Weddell Sea water masses involved in deep-water formation was highly similar, both at the molecular formula level (FT-ICR-MS; Fig. 2, 3) and in structural characteristics (^{1}H-NMR; Fig. 4). For FT-ICR-MS and ^{1}H-NMR, samples had to be concentrated and desalted. Solid-phase extraction (SPE) with modified styrene divinyl benzene polymer sorbents (PPL) is the most efficient extraction method to date [19,35]. Our SPE-DOC recovery (34%; Fig. 2A; Table S2) was within the range of previous SPE extraction efficiencies for Antarctic seawater DOC (14 – 42% [20,21]). Compared to total DOM, SPE-DOM analytical window selectively captures low molecular weight DOM [36] likely enriched in semi-labile and recalcitrant compounds [36,37], high degradation indices [21,37,38] and low organic nitrogen content [36]. Although some studies reported low $\Delta^{14}C_{SPE\text{-}DOC}$ values in SPE-DOM compared to total DOM [36,39,40], radiocarbon ages of bulk and SPE-extracted DOC were not significantly different in deep waters [36], and

bulk DOC and SPE-DOC behaved conservatively during deep-water mixing [11]. Moreover, the ^{14}C ages of DOC in the Southern Ocean (approximately 5200 – 5800 ^{14}C years [41] [42] [43]) are in the same range as those reported here for SPE-DOM (Fig. 2A, 4A). Taken together, previous observations suggest that the PPL-extracted fraction is broadly representative of the larger DOC pool, especially in the deep ocean. In our study, we focused on this fraction of DOM for molecular, isotopic and radiocarbon characterization and combined the SPE-DOM with physicochemical and DOC measurements obtained prior to the extraction.“

Specific comments:

Page 1

Title- I think the title not supported by the study, as only a fraction of DOM was characterized.

Response: Agree. We have rephrased the title (see general comment above) to (L1-2):

“Conserved molecular properties of dissolved organic matter during Weddell Sea Deep Water formation”

Line 29 “Therefore, carbon cycling processes, such as the production and turnover of dissolved organic matter (DOM), in AABW formation sites are crucial.” Not clear what is being said. Crucial how? to what?

Response: Agree. We have rephrased this sentence to (L28-31):

“Understanding the formation, degradation and export of dissolved organic matter (DOM) on the Antarctic continental shelf is therefore essential to determine how organic carbon enters the deep-sea reservoir.”

Line 32 That you are studying SPE DOM and that this is only a fraction of the total should be included in the abstract.

Response: Agree. We have added the SPE information in L33-34 and L37 of the Abstract.

Page 2

Line 69-70 “The spatial overlap…suggests carbon export to greater depths”. Not necessarily. It has been amply demonstrated that DOC concentration does not correlate with primary productivity. There is no reason to think productivity leads to export of DOC. Global sections of DOC concentration have no obvious component of export from the Southern Ocean into the deep Pacific. The uncertainty in the observational DOC data is too large to see a contribution if one exists.

Response: Agree. We have removed the sentence from this introductory paragraph and focus on carbon cycling and potential DOC export in the next paragraph (L73-89).

Line 76 “Dense water formation….deep-sea reservoir”. Not clear what is being said. All deep sea water and effectively all DOC originates from the surface. There may be small inputs of DOC from sediments in some places, but as far as we know these inputs are quantitatively negligible.
Response: Agree. We have rephrased it to (L73-75):
“At dense water formation sites, carbon cycling processes, such as the production, distribution and turnover of marine dissolved organic matter (DOM), are essential to understand how organic carbon enters the deep-sea reservoir”

Line 82 “unrecognizable biomolecules”. Readers may not know what you mean by this.
Response: Agree. We have rephrased the sentence to (L79-80):
“Marine DOC, as part of the dissolved organic matter (DOM) pool, is among the most complex organic mixtures known”.

Line 82 “DOM persistence…known biochemical features”. Again readers may not know what this sentence means, and it seems besides the point with regard to this paper.
Response: Agree. This sentence has been removed.

Line 84 “…its great reservoir size and long…”
Response: Agree. This sentence has been removed.

Line 84-96. It is not clear to me what the point of this paragraph is. I think it can be largely deleted.
Response: Agree. We have modified this paragraph considerably. We now include new text on how DOC can only be quantitatively relevant as an export term if it accumulates (L78-79) and the SPE-DOC average recovery in marine samples (L84-85).

Page 3
Line 146 “possible imprints on DOM pool size or reactivity”. I do not understand what is being said here. Please clarify. Are you saying you are looking for changes in DOM composition as the concentration of DOC changes?
Response: Agree. We have rephrased this sentence to clarify the study main objective and also highlight that we focused on the SPE fraction (L108-110):
“*In this study, we investigated DOM directly at the remote dense water formation sites on the southern shelf of the Weddell Sea, tracking the SPE-DOM composition along successive water mass transformation pathways.“*

Lines 146- “For FT-ICR-MS and 1H-NMR…prior to extraction (Fig.2; Table S2).” This sentence should go at the beginning of the paragraph (line 126), before any statement of property conservation so that the reader knows boundary conditions of the observations. It is important to include a statement that SPE captures < 50% of DOC.
Response: Agree. In the resubmitted manuscript, we have included a new paragraph on SPE (see general comment above and lines L127-145). We agree with the reviewer that the statement about SPE recovery should be included in the main text (it was only in the Methods L397 before). In the resubmitted version this information is now present in the introduction (L84-85) and in the results and discussion (L132-133).

Line 147 “Solid-phase extraction (SPE) with modified styrene divinyl benzene polymer sorbents (PPL) is the most efficient extraction method to date”. The relative efficiency of SPE is not this issue. The more important point is the amount of DOM SPE recovers (< 50% DOC). One could suggest that the fraction most likely to be impacted by biogeochemical processes, not the quantitatively most recoverable fraction, is the fraction of DOM to focus on here.
Response: Partially agree. We appreciate this important point. We have revised the manuscript to explicitly report SPE recovery and discuss the analytical window of SPE-DOM (see answer to major comment above). We have also clarified this by including the new subpanel in Fig. 2A with SPE-DOC concentrations per water mass, just below the DOC concentration, to make it clear to the reader.

Line 158 “Molecular formula data provide insights into the degradation state of Marine DOM.” I think the molecular degradation index developed by Fleurs (ref 37) does not provide any insight into DOM degradation. The radiocarbon old samples in the Fleurs paper show no differences in relative ion intensities. In this study the radiocarbon value between samples does not change (-510 per mil) and like the Fleurs paper, there is no change in molecular properties. But the issue of molecular degradation index aside, doesn’t this section repeat what has already been said in the preceding text, that vigorous mixing in the Southern Ocean homogenizes DOM enough that there is no changes in composition between the samples? I am not sure what the point of this discussion is.
Response: Partially agree. We thank the reviewer for raising this point. We agree that the molecular degradation index should not be interpreted as a direct measure of DOM degradation. Rather, I_{DEG} has been proposed as a compositional metric that correlates with radiocarbon age across diverse marine DOM samples (Flerus et al., 2012; *Biogeosci*). In our dataset, both $\Delta^{14}C$ values and I_{DEG} remained remarkably constant across the studied water masses, consistent with the broader conclusion of compositional homogeneity during deep-water formation. We have revised the text (L220-230) to clarify that I_{DEG} is presented as a complementary molecular descriptor related to ^{14}C, along two identified process-specific molecular indices (I_{BIO} and I_{PHOTO}) (L230-233). We also streamlined the discussion to emphasize how the comparison among FT-ICR-MS, ^{1}H-NMR, radiocarbon measurements, and I_{DEG} collectively supports the conclusion that DOM composition remains largely conserved across the investigated water masses.

Page 4

Line 190 “The typical decrease in DOM 14C-age in surface waters was absent in the Weddell Sea (Fig. 2A, 4A), indicating little to no net-input of modern carbon to DOM, despite the productive surface waters at the study site”. Are you making a distinction here between “radiocarbon” modern carbon and carbon “recently synthesized” by primary or secondary production? My recollection is that due to intense vertical mixing the radiocarbon value of DIC in the euphotic zone of the Southern Ocean is old (~ -260 per mil) – reflecting deep water values (I am thinking of the WOCE dataset). In that case “recently synthesized” DOC would not be “radiocarbon” modern, as it is in temperate and tropical waters where exchange with the atmosphere introduces bomb radiocarbon into the DIC, but instead have values of -200 to -300 per mil. If you are discussing “recently synthesized” carbon (and I think you are) then even a 10% contribution to DOM would fall within the 22 per mil uncertainty of the Δ14CSPE-DOC values, and could not be tracked with radiocarbon. This makes the subsequent discussion much less relevant.

Response: Partially agree. We thank the reviewer for this important clarification and agree that newly synthesized organic matter in the Southern Ocean may inherit the depleted $\Delta^{14}C$ signature of the dissolved inorganic carbon pool, which is influenced by the upwelling and mixing of old deep waters. Therefore, unlike in lower-latitude regions, the absence of a strong surface enrichment in $\Delta^{14}C$ does not necessarily imply an absence of recently produced DOM. We have revised the text accordingly and now emphasize that relatively modest differences in DOC $\Delta^{14}C$ are expected between surface and deep waters south of the Polar Front. However, our study combines radiocarbon measurements with molecular-level characterization of DOM. While newly produced DOM derived from aged DIC may not be readily distinguishable using $\Delta^{14}C$ alone, substantial accumulation of this material would be expected to alter the molecular composition of the DOM pool. The observed conservation of DOM molecular properties throughout the water column therefore suggests that recently produced DOM does not accumulate sufficiently to measurably modify the composition of the exported DOM pool. We have revised the discussion to clarify this distinction between isotopic and molecular indicators of DOM cycling (L189-202).

Line 203. This is to be expected, no change in DOC concentration, rapid mixing, deep waters to near the surface.

Response: Partially agree. We appreciate this perspective. Although the observed concentration may be expected from a mixing perspective, we keep this opening of the paragraph because it provides the necessary context for interpretation of the molecular results afterwards. To clarify that the result may be expected from a physical mixing perspective but still valuable to document empirically, we have added new text in the introduction (L98-106, see response to major comment above) and the term “surface mixing” in L265.

Page 5

Lines 220-235. I don’t understand the point of this discussion.

Response: Agree. We have removed this section. The corresponding space is used for the discussion on SPE which we agree is more relevant for our article (L127-145; see general comment above).

Line 237 “While the exact cause of the absence of signals from intense biological production in the deep-water masses is unknown”. See my general comments. I don’t think the authors have made a case as to why we would expect there to be a significant difference in the molecular composition of DOM between surface and deep waters here.

Response: Partially agree. We have removed this long paragraph. The way we present now the molecular information and absence of related signals to photodegradation and biodegradation is all combined in the paragraph in L213-240. There, we discuss the results from the I_{DEG}, I_{PHOTO} and I_{BIO} and provide context of our findings. We agree with the reviewer that the justification on potential differences in the molecular composition of DOM between surface and deep waters needed to be clarified and we have added corresponding text in L98-106 and L189-202.

Line 272 “DOM produced on the shelf was effectively removed likely due to rapid remineralization of labile DOM to inorganic carbon, enhanced by the long residence times of the deep and large Weddell Sea shelf 25. We suggest that the labile DOM produced at the surface became undetectable because it was rapidly transformed into refractory DOM 55, and this loss was not balanced by the production of new labile DOM in surface waters 11,30.”

I am unclear as to what is being said. The first sentence says that labile DOM is rapidly remineralized, while the second sentence says that labile DOM is undetectable because it is rapidly transformed into refractory DOM.

Response: Agree. In the resubmitted version of this article, we have included the possibility of labile DOM transformed into refractory DOM in a different paragraph (L256-257).

The technical aspects of the paper are fine. The sampling and analyses are all through established methods. The figures and captions are all well constructed and easy to follow.

Response: We would like to thank the reviewer for the positive comments on our manuscript. We believe that the input of both reviewers has significantly improved the quality of our resubmitted article.

Reviewer #1

The points raised by the first round of review appears to be incorporated in the revision. I support publication of the paper in Nature Communications.

Response: We thank Reviewer #1 for the positive assessment of our revised manuscript and for supporting its publication.

Reviewer #2

The authors have successfully revised the manuscript. I have some small additional comments below, some still related to my original review that the authors may have missed, and some points where I have no issue with what is being said, but where I think the text is not clear and could be improved.

Response: We thank Reviewer #2 for the constructive comments on our manuscript. We appreciate the suggestions, which have helped us further improve the clarity and quality of the manuscript.

Specific comments:

Lines 70-71. “mainly due….Ocean”. This should be deleted. It is not important to your point. There are major oceanographic expeditions to the Southern Ocean and Antarctic seas every year. For scientists in Europe, East Asia, and the US, access is not a problem. There are many reasons why scientists choose not to do this.

Response: Agree. The sentence has been removed. Thank you for the clarification.

Lines 87-89, isn’t the point you are trying to make is that although DOC concentrations suggest that Weddell Sea processing may not quantitatively impact the amount of carbon exported, changes in composition could impact the quality of that organic matter export. If this is your point, then the authors might replace the last sentence of the paragraph with a better summary than a statement that the Southern Ocean has been undersampled. (see my comment line 103)

Response: Agree. As suggested by the Reviewer, we have moved the sentence previously at line 103 to this section to clarify that changes in SPE-DOM composition may still affect the quality of exported organic matter (L85-88).

101 “In the Arctic Ocean…signature(32).” This sentence seems out of place. What does sea ice formation in the arctic have to do with this paragraph? The references cited studied the influence of sea ice formation on terrestrial organic matter, etc. which is quite different from the organic matter discussed here. This factoid and these studies

are not strong support for the case you are trying to make. One can stretch and try to connect the two, but it is a weak argument easily rebutted. It does not help your paper.

Response: Agree. The sentence about Arctic Ocean has been removed from the resubmitted version.

103 “Nevertheless….abyss”. This sentence seems to belong at the end of the preceding paragraph (line 89) where you make the case that composition may change without a change in concentration. I suggested on lines 87-89 that you write a better summary statement. This might be it.

Response: Agree. Thank you for the suggestion. We have moved and revised this sentence to conclude the previous paragraph (L85-88).

134 “the SPE-DOM analytical window”…

Response: Corrected as suggested.

141 “taken together…broadly representative…deep ocean” as I indicated in my initial review, there is strong evidence that the SPE-DOM fraction is NOT representative of the broader DOM pool. My impression from the author’s response was that they accepted this view.
As I pointed out last time, the high molecular weight DOM pool which is about the same size as the SPE-DOM pool, has a different radiocarbon (by several hundred per mil or several thousand years), stable isotope, and molecular composition than SPE-DOM. How is SPE-DOM representative of the HMWDOM pool? This statement and the supporting evidence that preceded it should be deleted. The paragraph should continue from line 136 directly onto line 143 (“nitrogen content (36). In our study we focused…”

Response: Agree. Thank you for the suggestion. In the resubmitted version, we have removed the statement implying that SPE-DOM is broadly representative, together with the related discussion.

Line 147 “Molecular properties of SPE-DOM were conserved…”

Response: Corrected as suggested.

Line 171 “Radiocarbon dating of SPE-DOM…”

Response: Corrected as suggested.

Line 178 "the youngest D14C(SPE-DOC) values…." (you use the "older" terminology elsewhere in the paragraph/paper. Since isotope values are negative numbers, this is the highest not the lowest value….)

Response: Revised as suggested.

Line 192 "…D14C values…" add a space

Response: Corrected as suggested.

Line 196-198. "This relatively small gradient reflects the upwelling of aged deep waters that supply an already old DOC pool to the Southern Ocean surface." This statement is true everywhere in the ocean, even where radiocarbon gradients are large. Upwelling of old DOC brings the apparent age of DOC in low latitude surface waters to about 2000 yrs. I suspect for most readers this will not be a clear explanation for the small gradient in radiocarbon. The small gradient is due the speed and intensity (depth) of the rapid vertical mixing of surface and deep water compared to low latitude regions (just as it does for nutrients, temperature, etc).

Response: Agree. Thank you for the suggestion. In the resubmitted version we have rephrased it to (L182-184): *"This relatively small gradient reflects the rapid vertical mixing of surface and deep waters compared to lower-latitude regions"*

Line 199 "due to the addition of modern radiocarbon into labile DOM" (or something like that, to distinguish radiocarbon from new production, which do not necessarily track each other).

Response: Revised as suggested.

Line 199 "The typical decrease in DOM 14C age, combined with no significant change in DOM composition was absent from the Weddell Sea". As written, this says there is a change in DOM composition with depth in the Weddell Sea ("no change" was absent). I don't think you mean that.
The text between lines 199-201 should be revised. The sentence infers that there are compositional differences between surface and deep waters in the Southern Ocean that are absent from the Weddell Sea. While you show compositional differences are absent from the Weddell Sea, I don't think you ever say or show explicitly that compositional differences with depth have been observed in the Southern Ocean.

Response: Agree. Thank you for the suggestion. We have revised the text to distinguish between the lack of a decrease in DOM ^{14}C age and the lack of significant changes in DOM composition. We have also removed the implication that depth-related changes are a general feature of the Southern Ocean (L185-189).

Line 204 “likely supported by….from remineralization”. I don’t understand the basis for this statement. What are the authors saying? The citation (as I remember it, it is for an old paper) attributes changes in carbon export to mineral ballasting. I don’t understand the connection to remineralization as discussed here and carbon export by sinking particles.

Response: Agree. We have removed this statement in the resubmitted version.

Line 207 “modern” is ambiguous. Do you mean labile DOC?

Response: Agree. We have replaced “modern” with “labile” DOC to clarify the meaning (L188, L191).

Line 225 “associated with the upwelling of deep waters”. I think you mean with the rapid vertical mixing of surface and deep waters since upwelling occurs everywhere (see above).

Response: Agree. We have rephrased it to (L209-211): *“This high degree of degradation is exceptional for DOM in surface waters and is likely associated with the rapid vertical mixing of surface and deep waters south of the Polar Front”*

Line 233 change “is related..” to “might be related..” since you do not really know why the signals are absent.

Response: Corrected as suggested.

Line 242-254. This paragraph could be deleted. I am not sure what the point of the paragraph is. The point about the patterns of primary production in the Weddell Sea and how they interact with DOC accumulation is opaque to the reader as is the sentence about autotrophic and heterotrophic production. I did not understand what the authors were trying to convey.

Response: Agree. The paragraph has been deleted. Thank you for the suggestion.

Line 247 Since we do not know the environmental conditions of the Weddell Sea in detail, change “Under the specific environmental conditions..” to “Under some conditions microheterotrophs can outcompete…”. The citation only says that if there is enough labile substrate, then heterotrophs can compete with autotrophs for iron. This is true of other nutrients, if there is enough substrate, which is always the issue….

Response: Agree. As suggested in the comment above, we have deleted this paragraph and removed this statement as well.

Line 258 “Our findings are in line with the most recent global oceanic DOC model suggesting rapid remineralization of labile DOC as a limiting factor controlling its contribution to long-term carbon storage.” This is saying that if DOM is rapidly remineralized it cannot contribute to long-term carbon storage. Isn’t that redundant?

Response: Agree. We have removed this sentence as recommended by the Reviewer in the following comment.

My own view is that lines 256-262 could be deleted as they do not add to the discussion. The point of the paragraph is from lines 262-268.

Response: Agree. We have removed it from the resubmitted version.

Line 281 change DOM to SPE-DOM

Response: Corrected as suggested.

Line 282 change DOM to SPE-DOM

Response: Corrected as suggested.

Line 298 change “on” to “in”. …dissolved organics in the open ocean….

Response: Corrected as suggested.
